# A plant-specific *HUA2-LIKE* (*HULK*) gene family in *Arabidopsis thaliana* is essential for development

**DOI:** 10.1111/tpj.12629

**Published:** 2014-08-28

**Authors:** Sathya S Jali, Sarah M Rosloski, Preetam Janakirama, Joshua G Steffen, Vladimir Zhurov, Thomas Berleth, Richard M Clark, Vojislava Grbic

**Affiliations:** 1 Department of Biology, Western UniversityLondon, ON, N6A 5B7, Canada; 2 Department of Biology, University of UtahSalt Lake City, UT, 84112, USA; 3 Center for Cell and Genome Science, University of UtahSalt Lake City, UT, 84112, USA; 4 Department of Cell and Systems Biology, University of TorontoToronto, ON, M5S 3B2, Canada

**Keywords:** *Arabidopsis thaliana*, gene family, phylogenetic analysis, redundancy, pleiotropic effects, lethality, transcriptomics

## Abstract

In *Arabidopsis thaliana*, the *HUA2* gene is required for proper expression of *FLOWERING LOCUS C* (*FLC*) and *AGAMOUS*, key regulators of flowering time and reproductive development, respectively. Although *HUA2* is broadly expressed, plants lacking *HUA2* function have only moderately reduced plant stature, leaf initiation rate and flowering time. To better understand *HUA2* activity, and to test whether redundancy with similar genes underlies the absence of strong phenotypes in *HUA2* mutant plants, we identified and subsequently characterized three additional *HUA2-LIKE* (*HULK*) genes in Arabidopsis. These genes form two clades (*HUA2*/*HULK1* and *HULK2*/*HULK3*), with members broadly conserved in both vascular and non-vascular plants, but not present outside the plant kingdom. Plants with progressively reduced *HULK* activity had increasingly severe developmental defects, and plants homozygous for loss-of-function mutations in all four *HULK* genes were not recovered. Multiple mutants displayed reproductive, embryonic and post-embryonic abnormalities, and provide detailed insights into the overlapping and unique functions of individual *HULK* genes. With regard to flowering time, opposing influences were apparent: *hua2 hulk1* plants were early-flowering, while *hulk2 hulk3* mutants were late-flowering, and *hua2* acted epistatically to cause early flowering in all combinations. Genome-wide expression profiling of mutant combinations using RNA-Seq revealed complex transcriptional changes in seedlings, with *FLC*, a known target of *HUA2*, among the most affected. Our studies, which include characterization of *HULK* expression patterns and subcellular localization, suggest that the *HULK* genes encode conserved nuclear factors with partially redundant but essential functions associated with diverse genetic pathways in plants.

## Introduction

Plants initiate primordia at the flank of the shoot apical meristem that give rise to leaves during vegetative development and to floral meristems during reproductive development. The primordial developmental fate is controlled by genetic pathways that integrate both endogenous and environmental cues, allowing plants to synchronize development with environmental conditions (Bernier *et al*., 1993; Telfer *et al*., 1997; Steynen *et al*., 2001; Bouyer *et al*., 2011). In *Arabidopsis thaliana*, timing of the transition to flowering depends on inputs from floral promoting and repressing pathways (Srikanth and Schmid, 2011). The major floral repressing pathway in Arabidopsis is controlled by *FLOWERING LOCUS C* (*FLC*). *FLC* represses the expression of floral pathway integrator genes that regulate the function of floral meristem identity genes. Combinatorial activities of floral meristem identity genes specify floral developmental fate to newly formed floral meristems (Bowman *et al*., 1991; Pelaz *et al*., 2000; Ditta *et al*., 2004; Pose *et al*., 2012). Among floral meristem identity genes, *AGAMOUS* (*AG*) specifies stamen and carpel identities and determinacy of the floral meristem (Bowman *et al*., 1991).

One gene that is implicated in the transition to flowering and floral developmental fate is *HUA2*. HUA2 is a putative transcription factor characterized by a PWWP domain that has roles in the epigenetic regulation of chromatin structure through interaction with histones and/or DNA, a RPR domain, that has been shown in yeast (*Saccharomyces cerevisiae*) to interact with splicing factors and/or the C–terminal regulatory domain of RNA polymerase II, and C–terminal PPLP repeats that are implicated in protein–protein interactions (Bedford *et al*., 1997; Kay *et al*., 2000; Steinmetz *et al*., 2001; Yang and Everett, 2007; Kang *et al*., 2009; Wang *et al*., 2009; Dhayalan *et al*., 2010). The combination of these domains suggests that HUA2 may couple transcription and pre-mRNA processing during mRNA synthesis (Montes *et al*., 2012). *HUA2* was initially described for its role in regulating *AG*, with Arabidopsis plants bearing a mutation in *HUA2*, in the *hua1 hua2 ag–4* mutant, displaying stamen to petal transformations, mis-shapen carpels and indeterminacy of the floral meristem (Chen and Meyerowitz, 1999). HUA1 is an RNA-binding protein, with six tandemly arrayed CCCH zinc fingers (Chen and Meyerowitz, 1999; Cheng *et al*., 2003). Features of both the HUA2 and HUA1 proteins suggest that the master C–function gene *AG* may be tethered to a RNA metabolism pathway.

As a single mutant, *hua2* plants develop normal flowers. Thus, the effect of *HUA2* on *AG* function is dependent on interacting loci (Chen and Meyerowitz, 1999), and subsequent genetic screens in the *hua1–1 hua2–1* background identified five *HUA ENHANCER* genes (*HEN1–5*) that also specify reproductive organ identity and floral determinacy. Several of these enhancers are known to function in diverse and seemingly unrelated pathways. For instance, HEN1 acts in microRNA metabolism (Boutet *et al*., 2003; Li *et al*., 2005), HEN3 encodes an E–type cyclin-dependent kinase (Wang and Chen, 2004), and *HEN5*/*PAUSED* (*PSD*) encodes a putative tRNA export receptor (Li and Chen, 2003). However, both HUA1 and HEN4 are RNA-binding proteins that interact *in vivo* (Li *et al*., 2001; Cheng *et al*., 2003), and *HEN2* encodes a putative RNA helicase (Western *et al*., 2002). Further, plants bearing *hua1 hua2* and *hen2* or *hen4* alleles were found to have elevated levels of aberrant *AG* transcripts that terminate within the large second intron, consistent with floral phenotypes associated with reduced *AG* activity (Cheng *et al*., 2003). This has led to the suggestion that *HUA2*, although lacking sequence homology to known RNA-binding proteins, may be associated with processing of *AG* pre-mRNA (Cheng *et al*., 2003).

Subsequently, independent studies also identified *HUA2* as a positive regulator of the floral repressors *FLC*, *MADS AFFECTING FLOWERING 1* and *2* (*MAF1* and *MAF2*) and *SHORT VEGETATIVE PHASE* (*SVP*) (Doyle *et al*., 2005; Wang *et al*., 2007). High *FLC* expression, which is characteristic of Arabidopsis strains that require exposure to prolonged cold in order to flower (i.e. are vernalization-dependent), is mediated by the activity of the *FRIGIDA* (*FRI*) locus that encodes a plant-specific protein (Johanson *et al*., 2000). FRI acts as a scaffold for assembly of a *FLC*-specific transcriptional activation complex that recruits general transcriptional machinery and chromatin modification factors (Choi *et al*., 2011). *HUA2* is required for both*FRI*-dependent and *FRI*-independent *FLC* expression, although the precise role of *HUA2* in regulating *FLC* expression has not been resolved (Doyle *et al*., 2005; Wang *et al*., 2007).

Despite findings linking HUA2 activity to expression of functional *AG* and *FLC* transcripts, the broader role of HUA2 (or HUA2-like) activity in plant development remained unclear. Although a lack of HUA2 activity has only modest effects on leaf initiation rate (Mendez-Vigo *et al*., 2010), plant stature and flowering time (Poduska *et al*., 2003; Doyle *et al*., 2005; Wang *et al*., 2007), and *HUA2*-associated floral phenotypes are only apparent in sensitized genetic backgrounds, the gene is nonetheless broadly expressed throughout development (Chen and Meyerowitz, 1999). Given the prevalence of gene duplications in the Arabidopsis genome, a consequence of paleopolypoidy events or more recent segmental or tandem duplications (Blanc *et al*., 2003), we wished to determine whether the absence of striking phenotypes in plants lacking *HUA2* may be explained by functional redundancy with related family members. Through homology searches, we identified a small gene family, the *HUA2-LIKE* (*HULK*) gene family, which consists of four members (including *HUA2*) in Arabidopsis. By constructing combinations of *HULK* mutants, we demonstrate that *HULK* gene function is essential for viability. The complex and sometimes opposing effects on development, flowering time and gene expression in mutant combinations suggest diverse and essential roles for *HULK* genes in the control of plant development and physiology.

## Results

### *In silico* identification of *HULK* genes

**Figure 1:**
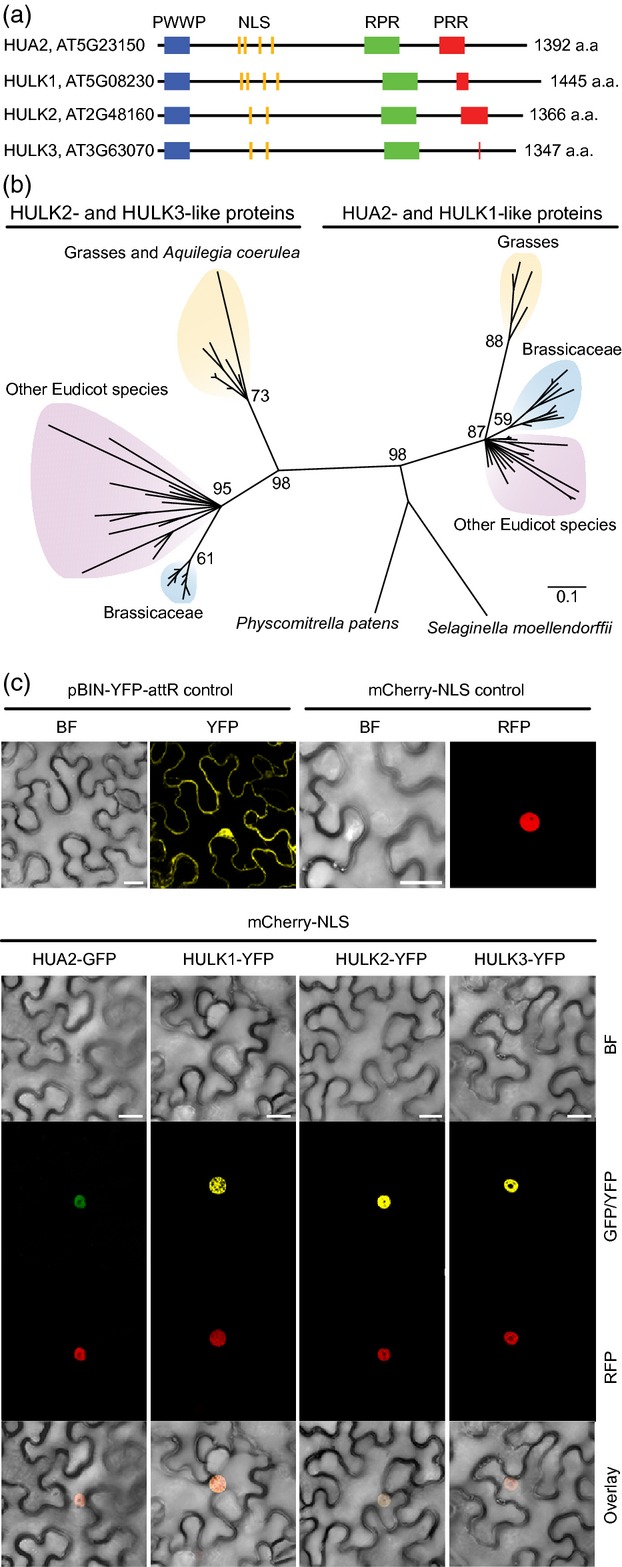
Structure of HUA2-like proteins of Arabidopsis and maximum-likelihood phylogeny of HULK-like proteins in Embryophytes.(a) Diagram of HUA2-like proteins of Arabidopsis, showing the PWWP, RPR and PRR domains and NLS motifs with relative positions and sizes.(b) Unrooted phylogram of amino acid sequences in domains of HULK-like proteins of 28 Embryophyte species, with *Physcomitrella patens* as an outgroup, based on maximum likelihood. The scale bar indicates the number of amino acid substitutions per site. Support values are puzzle support values.(c) Intracellular localization of HULK proteins. Top: confocal images of *N. benthamiana* leaf epidermal cells expressing the pBIN-YFP-attR or mCherry–NLS controls. Bottom: localization of co-infiltrated mCherry–NLS and HUA2–GFP, HULK1–YFP, HULK2–YFP or HULK3–YFP constructs, with an overlay of the bright field (BF), GFP/YFP and RFP images. Scale bars = 25 μm.

To identify genes with homology to *HUA2* in the Arabidopsis genome, we performed BLAST searches with *HUA2* nucleotide and protein sequences, and identified three genes that, together with *HUA2*, form the *HUA2-LIKE* (or *HULK*) gene family. The HULK family members share a conserved domain structure that includes a PWWP domain (Pfam: PF00855; named after the conserved Pro-Trp-Trp-Pro motif), putative nuclear localization signal (NLS) motifs (ELM: TRG_NLS_MonoCore_2), an RPR domain (SMART: SM000582; regulation of nuclear pre-mRNA) and a PRR domain (proline-rich region) of variable length (Figures1a and S1). Among the HULKs, pairwise amino acid identities range from 50.7 to 86.3% and from 47 to 88.6% for the PWWP and RPR domains, respectively. *HUA2* (AT5G23150) and *HULK1* (AT5G08230) are distantly linked on chromosome 5, while *HULK2* (AT2G48160) and *HULK3* (AT3G63070) are adjacent to the distal telomeres of chromosomes 2 and 3, respectively. Indicative of a comparatively recent gene family expansion, *HUA2*/*HULK1* and *HULK2*/*HULK3* are present within segmental duplications arising from the most recent paleopolyploidy event in the Arabidopsis lineage (Blanc *et al*., 2003).

A broader search for proteins with a similar domain organization to that in the HULK proteins retrieved sequences from plants within the sub-kingdom Embryophyta, but not in green algae, animals or fungi. Phylogenetic analyses using the PWWP and RPR domains of HULK homologs from evolutionary distant species revealed two well-supported clades represented by HUA2/HULK1 and by HULK2/HULK3 (Figure1b, puzzle support of 0.98). The split is ancient, and probably occurred in the common ancestor of angiosperms. With the exception of *Malus domestica*, *Brassica rapa* and *Populus trichocarpa*, all species for which we identified more than one HULK homolog have members belonging to both the HUA2/HULK1 and HULK2/HULK3 clades (Figure S2).

The presence of putative NLS motifs in each of the HULK proteins indicates that they may localize in the nucleus. To assess the subcellular protein localization of *HULK* gene products, we transiently expressed and visualized GFP/YFP-tagged proteins in tobacco epidermal cells (*Nicotiana benthamiana*). All four HULK proteins localized to the nucleus (Figure1c), a finding consistent with the presence of NLS motifs in each family member (Figure1a). Thus, we have identified a family of HULK proteins with broad phylogenetic distribution in the Embryophyta that localize in the nucleus.

### Expression of *HULK* gene family members

**Figure 2:**
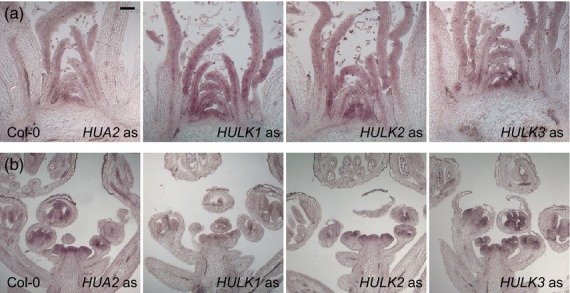
*HULK* gene expression in vegetative shoot and inflorescence apices as detected by *in situ* hybridization. Longitudinal sections of vegetative shoot (a) and inflorescence apices (b) hybridized with antisense probes to the four *HULK* genes as indicated. All sections are from wild-type (Col–0) plants. The sections were stained for 5, 7, 4 and 3 days, respectively, for *HUA2*, *HULK1*, *HULK2* and *HULK3* genes. Scale bar = 100 μm.

Based on both AtGenExpress (Schmid *et al*., 2005) and deep RNA-Seq read data from various tissues for 19 divergent accessions (see Experimental procedures and Gan *et al*., 2011), Arabidopsis *HULK* genes are expressed in all plant organs, as well as both vegetative and reproductive shoots (Figure S3). Although relative expression levels varied modestly by tissue, *HULK2* and *HULK3* were typically expressed more highly than *HUA2*, with *HULK1* having the lowest expression. To further characterize spatial expression patterns of each *HULK* gene, we performed *in situ* hybridization of both vegetative and reproductive apices of Arabidopsis using gene-specific probes. Consistent with known vegetative and reproductive phenotypes, and previous work demonstrating broad *HUA2* expression (Chen and Meyerowitz, 1999), we observed that *HUA2* was expressed throughout young primordia and vegetative and reproductive apices (Figures2 and S4). Likewise, each of the *HULK1–3* genes was expressed broadly in vegetative and reproductive apices in domains that are essentially indistinguishable from those for *HUA2* (Figure2). Although *in situ* hybridization is only semi-quantitative, expression of *HULK2* and *HULK3* occurred earlier than that of *HUA2* and *HULK1*, a finding that is consistent with the higher expression of *HULK2* and *HULK3* indicated by expression microarray and RNA-Seq data (Figure S3). Thus, *HULK* genes have overlapping expression patterns that, together with the commonality of the nuclear subcellular localization and high degree of sequence similarity of their corresponding gene products, suggest that they may act redundantly in performing their cellular functions.

### Characterization of *HULK* loss-of-function alleles

**Figure 3:**
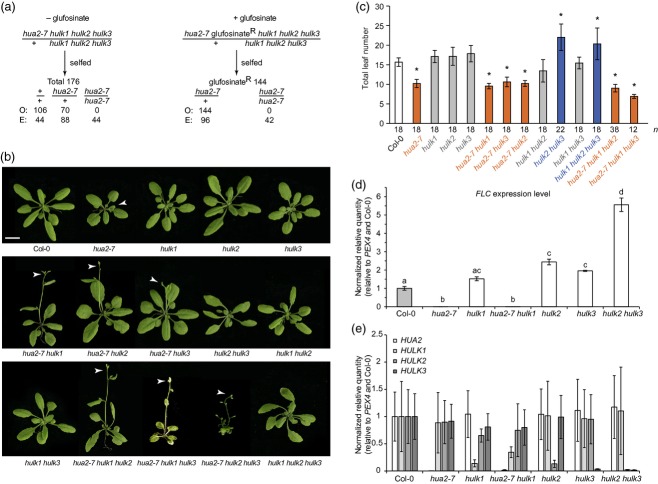
Genetic analysis of the quadruple *hulk* mutant, and phenotypes of single, double and triple *hulk* mutants.(a) Segregation analysis of *hua2–7*/*+ hulk1 hulk2 hulk3* progeny genotyped without glufosinate selection (left) and with glufosinate selection (right). ‘O’ and ‘E’ indicate the observed and expected numbers of plants of a given genotype, respectively.(b) Single, double and triple *hulk* mutants grown under a long-day photoperiod. Scale bar = 1 cm. All photographs are at the same magnification. Arrowheads indicate inflorescences.(c) Effect of *HULK* genes on flowering time. Flowering time is shown as the total leaf number ± sample standard deviation. The orange and blue bars indicate genotypes that flower earlier and later than Col–0, respectively (gray indicates no change). Asterisks indicate statistically significant differences in flowering time compared with Col–0 after correcting for multiple tests.(d, e) Expression levels of *FLC* (d) and *HULK* genes (e) in single and double mutants belonging to either the *HUA2*/*HULK1* or *HULK2*/*HULK3* clades. Values are means ± standard error for fold changes relative to Col–0 detected by quantitative RT–PCR (*n *=* *3). Different letters in (d) indicate HSD test groups at α = 0.05.

The presence of coding sequence disruptions (*hua2*, *hulk1* and *hulk3* mutants; Figure S5), reduced expression observed using quantitative RT–PCR and RNA-Seq (all *hulk* alleles; Figures3, S5 and S6, and Tables S1 and S2), the recessive behavior of each allele in crosses (see below), and the similarity of phenotypes induced by T–DNAs versus amiRNAs (Figures S7 and S8) suggest that all the alleles included in our study are loss-of-function alleles; however, they may not all be null, and *hulk2* is likely hypomorphic (Figures S5 and S6).

### *HULK* genes affect flowering time and are essential for normal development

To assess the roles of *HULK* genes during development, we characterized plants homozygous for single, double or triple *HULK* mutations. Although we attempted to create a quadruple mutant, we were unsuccessful. Analysis of the progeny of selfed *hua2–7*/*+ hulk1 hulk2 hulk3* plants that were selected for glufosinate resistance linked to the *hua2–7* allele resulted in the exclusive retrieval of plants heterozygous for *hua2–7* (Figure3a). Further, of 176 genotyped progeny of *hua2–7*/*+ hulk1 hulk2 hulk3* self-pollinated plants, none were homozygous for the *hua2–7* allele (Figure3a). This deviates significantly from the expected 1:2:1 ratio (χ^2^ = 135.045, *P *<* *0.0001), and *+*/*+ hulk1 hulk2 hulk3* plants were actually over-represented. It remains to be determined whether the inactivation of all *HULK* family members precludes gamete formation, fertilization, or development of the zygote.

Although the gene family is essential for development, substantial functional redundancy among *HULK* genes was apparent (Figure3b). Single and double mutant combinations displayed normal or near-normal plant morphology, with pronounced defects in shoot size and morphology apparent only in some triple mutants. We have previously shown that *hua2–7* mutants flower earlier than wild-type, as measured by the reduced number of rosette leaves initiated during vegetative development prior to the transition to flowering (Wang *et al*., 2007). To further assess the role of *HULK1–3* in the regulation of flowering time, we recorded the leaf number at flowering of wild-type and single, double and most triple mutants (Figure3c); the developmental abnormalities of *hua2–7 hulk2 hulk3* mutant plants and their frequent lethality prior to reaching the reproductive stage prevented analysis of the flowering time in this genotype. anova demonstrated a significant effect on flowering time for each *HULK* gene as a factor (*P *<* *4.3 × 10^−6^). We also found that the interaction term was highly significant (*P *< 10^−15^), indicating that the effect of the *HULK* genes on flowering time was non-additive (i.e. epistasis was apparent). Overall, the presence of *hua2–7* was sufficient to accelerate flowering in any mutant combination that was assessed for flowering time.

To examine the effects in specific contrasts, we performed pairwise comparisons among all genotypes included in our anova analysis. After adjusting *P* values using the Tukey–Kramer method to control for experiment-wide multiple testing, we found that eight genotypes differed significantly from wild-type in terms of flowering time (*P *< 10^−7^ in all cases; indicated by asterisks in Figure3c). All single, double and triple mutant combinations that lacked *HUA2* function flowered earlier by approximately five leaves. Although single mutants in *HULK1–3* did not differ significantly from wild-type in terms of flowering time in pairwise tests, two mutant combinations with wild-type *HUA2* activity, but lacking *HULK2* and *HULK3* function (*hulk2 hulk3* and *hulk1 hulk2 hulk3*), flowered later than wild-type by approximately five leaves.

Previous studies established that *HUA2* is required for maintaining expression of the floral repressor *FLC* (Poduska *et al*., 2003; Doyle *et al*., 2005; Wang *et al*., 2007). To examine whether *HULK* genes affect *FLC* expression, we determined *FLC* transcript levels in *hua2/hulk1* and *hulk2/hulk3* single and double mutants by quantitative RT–PCR. As shown in Figure3(d), *FLC* transcript levels correlated with the number of rosette leaves produced. For example, *FLC* levels were reduced in *hua2–7* and *hua2–7 hulk1* plants, but were increased in the *hulk2 hulk3* mutant. These data suggest that *HULK* genes have antagonistic effects on the regulation of flowering. To exclude the possibility that potential compensatory regulation among *HULK* genes may account for the antagonistic effects on flowering time and *FLC* expression, we determined the transcript levels for each *HULK* gene in single and clade-specific double mutant backgrounds. As shown in Figure3(e), mutation in any one of the *HULK* genes has no effect on expression of wild-type alleles of the remainder of the family members, indicating that *HUA2* and *HULK2*/*HULK3* act independently as a repressor or accelerators of flowering, respectively. However, the *hua2–7* allele is epistatic to other *hulk* alleles in terms of regulation of flowering time. The action of these loci is consistent with modulation of the expression of *FLC* (the flowering time master repressor). However, their mechanism(s) of action is (are) presently unknown.

### Unique and overlapping functions of individual *HULK* genes

Analysis of multiple *hulk* mutants revealed a complexity of unique and overlapping gene functions, which largely reflected the phylogenetic relationship of the *HULK* genes. Most developmental functions were redundantly performed by the two members within each clade, but overlapping functions across clades are indicated by further enhancement of certain traits in some triple mutants and the lethality of the quadruple mutant.

### Disruption of the *HUA2/HULK1* clade

**Figure 4:**
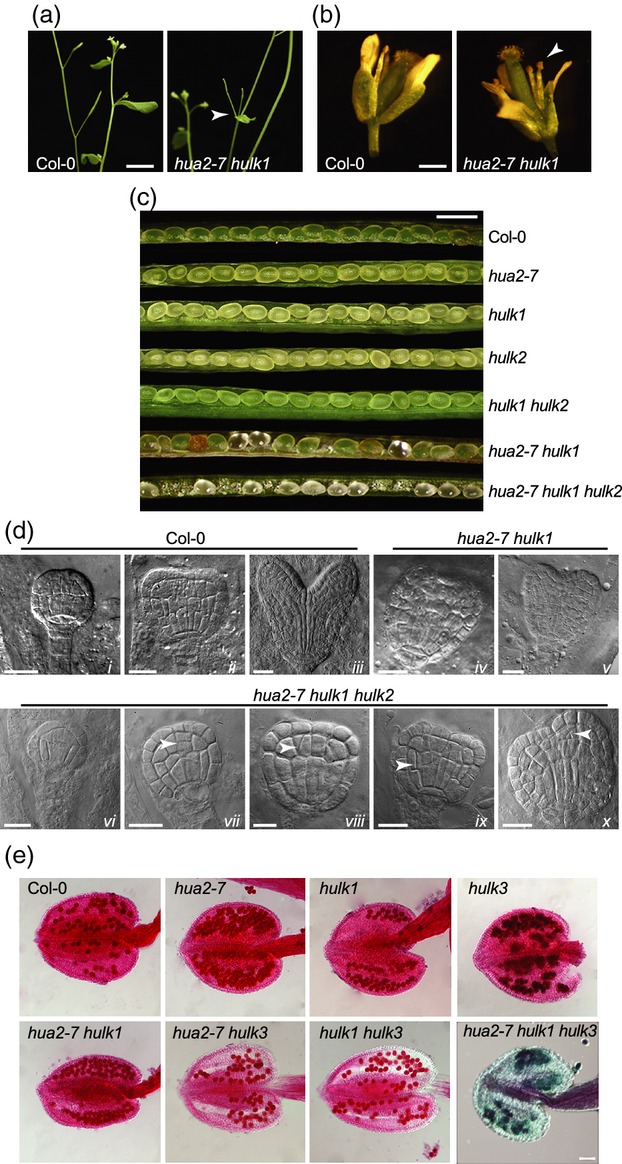
Morphological characterization of single, double and compound triple mutants lacking *hua2-7* and/or *hulk1* activities.(a) Adult Col–0 wild-type and *hua2–7 hulk1* plants. The arrowhead indicates a *hua2–7 hulk1* inflorescence with two undeveloped siliques at the stem node.(b) Col–0 wild-type and *hua2–7 hulk1* flowers. The stamens (arrowhead) are shorter than the stigma in mutant plants.(c) Col–0 and mutant siliques collected from the middle of the inflorescence.(d) Nomarski images of Col–0 embryos at the early globular, triangular and mid-heart stages (i, ii and iii, respectively), and arrested embryos in double mutant *hua2–7 hulk1* embryos (iv and v) and triple mutant *hua2–7 hulk1 hulk2* embryos (vi–x). Note the normal cell shape, even in very sensitive epidermal cells, and normal tissue layers and organ initiation in mutant embryos. Mutants may show variant cell numbers in diverse parts of the embryo (arrowheads).(e) Col–0 and mutant stamens stained with the Alexander stain. Viable pollen stains deep pink, while *hua2–7 hulk1 hulk3* pollen stains green/blue, indicating lack of viability. Scale bars = 1 cm (a), 1 mm (b), 20 μm (d) and 50 μm (e).

Although there were no obvious changes in basic plant growth in *hua2–7 hulk1* plants, there were sporadic alterations in flower phyllotaxy (Figure4a) that were not seen in single *hua2–7* or *hulk1* mutants, but were previously reported in *hen2 hua2* plants (Western *et al*., 2002). Further, early flowers in *hua2–7 hulk1* plants were sterile, observed as undeveloped siliques due to asynchronous growth between stamens and pistils (Figure4a,b). When siliques with seed were produced, the seeds in *hua2–7 hulk1* plants, but not in *hua2–7* and *hulk1* single mutants, had disrupted development (Figure4c). For siliques collected from the middle of the inflorescence, the embryos of approximately 67% of seeds (*n *=* *185) showed developmental arrest at various stages relative to normally developing seed within the same silique and the corresponding wild-type (Figures4c and S7). A similar wide range of seed developmental arrest has been described previously for several mutants in genes required for normal seed development, such as *oma–1*, *fey–1* and *ila–1* (Johnston *et al*., 2007). In the case of *hua2–7 hulk1*, the defective embryo phenotype progressively diminished during reproductive development, such that siliques toward the top of the inflorescence appear normal. The embryo defects seen in *hua2–7 hulk1* plants were enhanced by loss of *HULK2* function. In *hua2–7 hulk1 hulk2* triple mutant plants, the defective embryo phenotype had high penetrance, and the plants uniformly showed earlier stages of seed arrest compared with phenotypes observed in *hua2–7 hulk1* plants. In siliques collected from the middle of the inflorescence, all seeds were translucent, with embryos that were arrested in early development, in contrast to normal seed development in *hulk2* and *hulk1 hulk2* plants (Figure4c). Inspection of the translucent seeds of both *hua2–7 hulk1* and *hua2–7 hulk1 hulk2* plants showed that embryos arrested in the globular to early heart stage of development (Figure4d). Individual embryo cells were of normal shape and had a normal cell-wall thickness, and incipient tissue layers and seedling organs were established in the expected locations. Instead, defects in mutant embryos appear to comprise heterogeneous and random changes in cell numbers in various parts of the embryo. In the context of characterized embryo-lethal mutants (Johnston *et al*., 2007; Meinke *et al*., 2008), such defects suggest that *HULK*s may affect cell proliferation either through its regulation or by providing a limiting metabolic compound.

**Figure 5:**
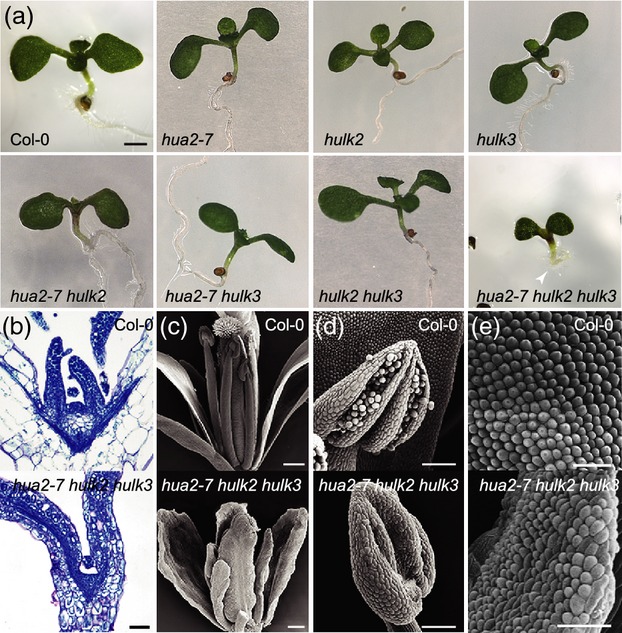
Morphological characterization of the *hua2–7 hulk2 hulk3* triple mutant.(a) Morphology of 9-day-old wild-type Col–0 and mutant seedlings. The arrowhead indicates roots with arrested development in the *hua2–7 hulk2 hulk3* mutant.(b) Longitudinal sections of 9-day-old wild-type Col–0 and *hua2–7 hulk2 hulk3* seedlings showing shoot meristem abnormalities in the mutant.(c) Scanning electron micrographs of Col–0 and *hua2–7 hulk2 hulk3* flowers.(d) Scanning electron micrographs of stamens of wild-type Col–0 and *hua2–7 hulk2 hulk3*.(e) Scanning electron micrographs of Col–0 petal cells and *hua2–7 hulk2 hulk3* petaloid stamen cells. Scale bars = 20 μm (b), 150 μm (c), 100 μm (d) and 50 μm (e).

Although HULK2 and HULK3 share a high degree of protein sequence similarity, loss of *HULK3* function in the sensitized *hua2–7 hulk1* background resulted in a different phenotype relative to *hua2-7 hulk1 hulk2*. Triple mutant *hua2–7 hulk1 hulk3* plants were reduced in size with small leaves and only a few branches (Figure3b); additionally, these plants were entirely sterile due to pollen inviability (green/blue coloration as observed with the Alexander stain, see Figure4e), and triple mutant plants had to be derived from heterozygotes at the *HUA2* locus (i.e. *hua2–7*/*+ hulk1 hulk3* plants). Single mutant plants (*hua2–1*, *hulk1* and *hulk3*) and the double mutants *hua2–7 hulk1* and *hua2–7 hulk3* all showed normal pollen viability (Figure5e). Thus, *HUA2* and *HULK1* act redundantly to affect shoot phyllotaxy, stamen outgrowth in early flowers and embryo development. *HULK2* acts redundantly with *HUA2* and *HULK1* in embryo development, while *HULK3* function is required for pollen development in the *hua2–7 hulk1* mutant background.

### Disruption of the *HULK2/HULK3* clade

Loss of function of the *HULK2*/*HULK3* clade in *hulk2 hulk3* mutant plants resulted in normally developing but late-flowering plants (Figure3). Further loss of *HULK1* function in this sensitized background had no visible effect on plant development, and *hulk1 hulk2 hulk3* mutant plants recapitulated the late-flowering phenotype seen in *hulk2 hulk3* mutants (Figure3c). By contrast, the effect of loss of *HUA2* function in the *hulk2 hulk3* background was dramatic (Figure5). Triple *hua2–7 hulk2 hulk3* seedlings were smaller than wild-type and had aberrant root development (Figure5a). Few triple mutants survived on soil, and those that survived were stunted and often failed to induce reproductive development and bolt (Figure3b). As assessed by serial longitudinal sections of the vegetative meristem, *hua2–7 hulk2 hulk3* mutants had a flattened shoot apical meristem with an abnormal and reduced rib zone, as opposed to the wild-type shoot apical meristem with a high central dome on top of an expanded rib zone comprising well-defined large, round and lightly stained cells (Figure5b). In addition, mutants displayed randomly initiated and mis-shapen leaf primordia that developed into shoots with abnormal phyllotaxis and abnormal leaf shapes (Figure3b). When reproductive development was initiated in *hua2–7 hulk2 hulk3* plants, the reproductive tissues were abnormal, as assessed by scanning electron microscopy (Figure5c–e). Petals in early flowers were often absent and stamens were aberrant, showing sterility, failure to dehisce, and occasionally a petaloid morphology. Later flowers showed more defined stamen structures, although neither early nor late flowers produced fertile stamens. In early flowers, the gynoecium was mis-shapen with disorganized stigmatic hairs (Figure5c). Cross-pollination of flowers with wild-type pollen did not result in fertile siliques, suggesting aberrant pollen tube germination or guidance, development of defective female gametophytes, or development of defective integuments. Although there was a spectrum of severity in flower defects, none of the flowers displayed completely normal morphology, and this mutant line was maintained as a *hua2–7 hulk2 hulk3*/*+* stock. Thus, *HUA2* and *HULK1* have different effects in the *hulk2 hulk3* mutant background: while the function of *HULK1* seems dispensable, *HUA2* function is required redundantly with *HULK2* and *HULK3* for establishment of normal plant morphology.

### Expression profiling of *HULK* mutants

To further investigate the roles of *HULK* genes, we performed transcriptome profiling using Illumina RNA-Seq with biological replication for seedlings of Col–0, *hua2–7* single mutants, *hua2–7 hulk1* double mutants and *hua2–7 hulk1 hulk2* triple mutants grown at 20°C under long-day conditions (16 h light). To compare gene expression, we selected seedlings at similar developmental stages (emergence of the 4th true leaf) and used whole aerial shoot tissue for RNA preparation. For the triple mutant, only a subset of progeny were collected owing to reduced germination and developmental asynchrony, an inherent experimental limitation that potentially biased the resultant triple mutant transcriptome profiles toward wild-type. By aligning resulting RNA-Seq reads to the Arabidopsis genome, we calculated normalized expression levels for all genes in the latest annotation (TAIR10, http://www.arabidopsis.org/). On average, we aligned 3.5 million 78 bp reads per replicate (Table S1). In every case, the Pearson *R*^2^ value for coding gene expression levels between biological replicates exceeded 0.99 (Figure S9). On average, approximately 17 400 genes per genotype were found to be expressed (five or more reads mapped), a finding similar to that reported in an experimentally comparable study that used seedlings of wild accessions (Table S2) (Gan *et al*., 2011).

**Figure 6:**
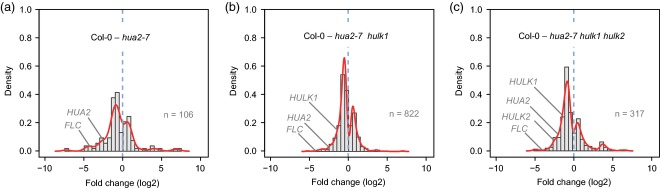
Analysis of differential gene expression in mutant combinations. Histograms of fold changes for genes differentially expressed between Col–0 and the *hua2–7* single mutant (a), the *hua2–7 hulk1* double mutant (b), and the *hua2–7 hulk1 hulk2* triple mutant (c). For each comparison, the number (*n*) of differentially expressed genes is shown (false discovery rate of 5%). In all cases, *HULK* genes and *FLC* are indicated if they were detected as differentially expressed in a given comparison. To facilitate display, red lines show smoothed densities.

We identified genes with differential expression compared to wild-type (Col–0) using the DESeq package (Anders and Huber, 2010), and detected 106, 822 and 317 genes in the single, double and triple mutants, respectively, at a false discovery rate of 0.05 (see Experimental Procedures). Among these, genes with reduced expression relative to wild-type were observed more often (Fisher's exact test, *P *<* *0.05 in each comparison). However, the majority of differently expressed genes had low fold changes, and only 53/106, 235/822 and 124/317 differentially expressed genes were more than twofold up- or down-regulated in the single, double and triple mutants, respectively (Table S3). Although most differentially expressed genes showed only modest fold changes, *FLC* was either the most strongly down-regulated gene, or one of the most strongly down-regulated genes, in the single, double and triple mutants (Figure6 and Tables S2 and S3). Thus, compared to all genes, the extent to which *FLC* is down-regulated in mutant combinations lacking *hua2* function is exceptional, and is associated with the uniformly early-flowering phenotype in the various mutants (Figure3). In addition to *FLC*, several other genes with known roles in flowering time regulation displayed decreased expression levels, such as *MAF1* in double and triple mutants, and *TEM1* and *TEM2* in single, double and triple mutants (Table S2).

Gene ontology (GO) enrichment analysis of differentially expressed genes showed an over-representation of genes associated with responses to biotic and abiotic stimuli, and reproductive and post-embryonic development, as well as transcription factor or DNA- binding activities. Table S4 provides a complete list of enriched GO terms and associated genes; the top 20 GO functional categories in each mutant showed a strong overlap (Figure S10). The diversity of GO categories may reflect the pleiotropic nature of the double and triple *hulk* mutant combinations. Further, loss-of-function phenotypes associated with the differentially expressed genes (Lloyd and Meinke, 2012) may contribute to the defects in phyllotaxy (*IBM1*) and the reduced growth (*EXO*, *MLS*, *IBM1*, *MAF1* and *FLC*), fertility (*DRL1*, *IBM1* and *TKPR1*) and seed viability (*SPK1*) observed in compound *hulk* mutants. In addition, differentially expressed genes were enriched for transcription factors belonging to the AP2/ERF superfamily (Table S5). The effect of the HULK proteins on AP2/ERF superfamily expression and the similarity of AP2/ERF developmental functions to those of the HULK proteins, i.e. flower, embryo and root development, meristem determinacy and epidermal cell identity, as well as the response to abiotic and biotic stimuli, suggests these two gene families may have a functional association (Elliott *et al*., 1996; Moose and Sisco, 1996; Chuck *et al*., 1998; Liu *et al*., 1998; Boutilier *et al*., 2002; Gu *et al*., 2002; Hirota *et al*., 2007).

The RNA-Seq data revealed that *hulk* genes included in respective mutant combinations were down-regulated, an expected finding given the characterized T–DNA disruptions (Figures5 and S4). However, we found no evidence to suggest compensatory up-regulation of unaffected (non-mutant) *HULK* transcripts as an explanation for our finding of functional redundancy among family members, i.e. *HULK1–3* were not up-regulated in the *hua2–7* single mutant, and *HULK3* was not up-regulated in the *hua2–7 hulk1 hulk2* triple mutant (Figure6).

## Discussion

The HULK proteins of Arabidopsis share high sequence similarity in conserved domains, and each has a clear ortholog in several related diploid Brassicaceae species (e.g. the congener *Arabidopsis lyrata* and *Capsella rubella*). Among these, phylogenetic analyses revealed two well-supported clades consisting of *HUA2*/*HULK1* and *HULK2*/*HULK3*, a finding consistent with an analysis of all PWWP domain proteins in Arabidopsis species that included the HULK family members (Alvarez-Venegas and Avramova, 2012). In our broader survey of available plant genomes, we found that *HULK* genes have been ubiquitously retained from the ancestor of vascular and non-vascular plants. However, they are not found outside the Embryophyta. Across represented taxa, *HULK* orthologs bear similarity to either the *HUA2*/*HULK1* clade or the *HULK2*/*HULK3* clade, consistent with a basal duplication event, potential diversification of function, and widespread retention of a small number of members (approximately one to three genes) in each clade.

In agreement with their broad phylogenetic distribution in plants, a pattern typical of genes that perform essential functions, Arabidopsis plants homozygous for loss-of-function mutations in all *HULK* family members were not recovered, and thus the family is required for plant development. Nevertheless, significant functional redundancy was apparent. Although *hulk1*, *hulk2* and *hulk3* single mutants did not have obvious phenotypes, double and triple *hulk* mutants were increasingly affected. The failure to identify *HULK1–3* in laboratory mutagenesis screens therefore probably resulted from redundancy (Lloyd and Meinke, 2012). However, in contrast to many duplicate genes in Arabidopsis, *HULK1–3* do not segregate for obvious loss-of-function mutations in population samples used in recent high-throughput re-sequencing studies (Cao *et al*., 2011; Gan *et al*., 2011). Whether *HULK1–3* are required individually for wild-type fitness in natural settings is unknown. Among double mutants, the strongest phenotypes were observed when both members of either the *HUA2*/*HULK1* or the *HULK2*/*HULK3* clades were inactivated. This suggests stronger redundancy within clades than between clades, as has been observed in studies of other gene duplicates (e.g. Hanada *et al*., 2011). The asymmetric redundancy seen between *HUA2* and *HULK1* (e.g. the mutant phenotypes observed in *hua2 hulk2 hulk3*, but not in *hulk1 hulk2 hulk3*) may be a reflection of different levels of expression of the two genes (Figure S3). Nevertheless, the dramatic phenotypes observed in triple and quadruple mutants revealed functional redundancy across the two *HULK* clades despite their ancient origin (a gene duplication event at the base of Embryophyta). In triple mutants, phenotypic consequences were most notable when *HUA2* was inactivated. Conversely, plants retaining both or only one active copy of *HUA2* with loss of the rest of the gene family were phenotypically normal. Thus, with respect to the phenotypes we examined, *HUA2* plays a more prominent role in Arabidopsis.

Plant stunting, effects on leaf and silique phyllotaxy, loss of pollen viability and/or repressed stamen maturity, petaloid stamens and embryo abortion were previously observed in plants that lack *HUA2* function in genetic backgrounds with compromised function of *HUA1* and *HEN1–4* genes (Chen and Meyerowitz, 1999; Western *et al*., 2002; Cheng *et al*., 2003; Wang and Chen, 2004). The developmental defects in compound *HULK* mutants suggest roles for *HULK* genes in many of these processes. For instance, triple mutants bearing *hua2* or *hulk3* alleles were stunted, similarly to phenotypes reported in *hua2 hen4* and *hua1 hua2 hen1* plants (Chen and Meyerowitz, 1999; Chen *et al*., 2002; Cheng *et al*., 2003). Loss-of-function mutants of *HUA ENHANCER 3*, an E–type cyclin-dependant kinase, had very small leaves, similar to the *hua2 hulk1 hulk3* and *hua2 hulk2 hulk3* mutants in our study (Wang and Chen, 2004). The altered phyllotaxy of siliques in *hua2–7 hulk1* plants was also observed in *hua2 hen2* plants, suggesting that either loss of *HEN2* or *HULK1* may be sufficient to disrupt silique phyllotaxy in the *hua2* background (Western *et al*., 2002). Loss of pollen viability and/or repressed stamen maturity observed in *hua2 hulk1*, *hua2 hulk2 hulk3* and *hua2 hulk1 hulk3* plants was also reported for *hua2 hen2* plants (Western *et al*., 2002). *HULK1–3* are therefore likely to act, together with *HUA2*, in diverse developmental pathways that had until now been associated with *HUA2* activity in sensitized backgrounds harboring mutations in genes encoding unrelated proteins.

The pleiotropic nature of defects in *hulk* mutants is consistent with expression profiling that revealed effects of *HULK* members on the expression levels of hundreds of genes, many of which have known developmental roles. Despite the large number of differentially expressed genes, the fold changes in seedlings were usually moderate, although effects on expression may be more dramatic during developmental stages that are strongly affected in the mutants (e.g. embryos and flowers). As in expression of hundreds of genes was altered, the basis for differential gene expression is probably indirect in many cases. However, as *HUA2* has been linked to the activation or maintenance of gene expression (Chen and Meyerowitz, 1999; Western *et al*., 2002; Doyle *et al*., 2005; Wang *et al*., 2007), our finding that down-regulated genes were over-represented raises the possibility that an appreciable fraction of the differentially expressed genes that we detected are direct HULK targets.

The effects of the *hulk* mutations on flowering time and *FLC* expression provide insights into the functional relationship among *HULK* genes. First, the knockout of two clades had opposite effects: *hua2 hulk1* mutants flowered early, while *hulk2 hulk3* mutants flowered late. Second, in all mutant combinations lacking *HUA2* activity, flowering time was reduced. Third, all flowering time effects were reflected in our quantitative RT–PCR and expression profiling experiments, in which *FLC* was among the most strongly and uniformly down-regulated genes in mutant combinations lacking *HUA2* function; consistently, *FLC* levels were up-regulated in the *hulk2 hulk3* mutant. These results indicate that the *HULK* genes affect *FLC* expression in a gene and clade-specific manner, with *HUA2* and *HULK2*/*HULK3* acting antagonistically on *FLC* expression and flowering time. The epistatic nature of the effect of *HUA2* on flowering time may be explained by a non-redundant role for *HUA2* in regulating *FLC* expression.

The prominent role of *HUA2* within the *HUA2*/*HULK1* clade was also observed in virtually all other processes, and may reflect a case of asymmetric redundancy (Briggs *et al*., 2006). The dramatically lower expression levels of *HULK1* relative to *HUA2* probably contribute to the observed asymmetric redundancy; however, a critical role for *HULK1* is apparent in sustaining embryo development, which is arrested in *hua2–7 hulk1* and *hua2–7 hulk1 hulk2*, but not in *hua2–7 hulk2* plants (Figure4). In contrast to their antagonistic roles in controlling flowering time, *HUA2* and *HULK2*/*HULK3* genes act redundantly in all other aspects of development (Figure5). The triple mutant *hua2–7 hulk2 hulk3* displays marked alterations in a number of developmental processes, which were not observed in any double mutant combination of these alleles. Loss of either *HUA2* or the *HUA2*/*HULK1* clade function also allowed identification of specific roles for *HULK2* and *HULK3*. For example, *HULK3* acted redundantly with *HUA2* and *HULK1* to promote pollen development (consistent with high levels of *HULK3* in pollen, Figure S3), while *HULK2* contributed to promoting embryo development in the same background (Figure4). More generally, our findings suggest a model whereby *HULK* genes act redundantly to regulate a subset of essential genes, with some (or all) family members also having specific functions. Our finding that *HULK* genes are expressed in overlapping expression domains and that their functional relationships largely reflect their protein sequence similarities suggests functional divergence at the protein level. Future work is required to understand the biochemical action of *HULK* genes, and to resolve mechanism(s) of *HULK* gene activities.

## Experimental Procedures

### Phylogenetic analysis of *HUA2-LIKE* sequences in Embryophyta

Identification of genes with homology to *HUA2* in the *A*. *thaliana* genome, retrieval of Embryophyta gene families (Table S6), and processing of protein sequences prior to phylogenetic analysis are described in Methods S1 (Castresana, 2000; Edgar, 2004; Goodstein *et al*., 2012). A maximum likelihood-based phylogenetic tree was generated using tree-puzzle (http://www.tree-puzzle.de/) version 5.2 software with the following parameters: number of puzzling steps 100 000, model of substitution JTT+G8 + I with automatic estimation of α, and model parameter estimation using quartet sampling + NJ tree (Jones *et al*., 1992; Schmidt *et al*., 2002). The resulting tree was visualized using Dendroscope (Huson *et al*., 2007) with *Physcomitrella patens* being used as an outgroup. Branches with puzzle support values below 50% were collapsed.

### Plant materials and growth conditions

All Arabidopsis mutants described in this study are in the Columbia (Col–0) background. The T–DNA insertion allele for *hua2–7*, carrying a glufosinate resistance gene, has been described previously (Wang *et al*., 2007). The T–DNA insertion alleles for *hulk2* (SALK_029629) and *hulk3* (SALK_072659) were obtained from Arabidopsis Biological Resource Center (Alonso *et al*., 2003). *hulk2* plants are kanamycin-resistant, but the resistance gene is silenced in *hulk3* plants. The *hulk1* seed was obtained from GABI-Kat (GK156C05) (Kleinboelting *et al*., 2012). *hulk1* seedlings are resistant to sulfadiazine. Seeds were surface-sterilized and stratified at 4°C for 2–4 days. Plants were grown at 20–22°C, under either long days (16 h light/8 h dark) or short days (8 h light/16 h dark), 65% relative humidity under a 2:1 mixture of cool white and warm light at an irradiance of 125–175 μmol m^−2^ sec^−1^. For flowering time analyses, leaf number was counted from at least 12 plants per genotype.

### Generation of mutants defective in *HULK* genes

Double mutants of *HULK* gene members were created by crossing single mutants in all possible combinations. The resulting F_2_ populations were PCR-genotyped for homozygous double mutants using the primers listed in Table S7. The *hua2–7 hulk2 hulk3* triple mutant was generated from a cross between a *hulk3* female parent and a *hua2–7 hulk2* male parent. Because *hua2–7 hulk2 hulk3* mutants are sterile, they were maintained as heterozygous plants at the *HULK3* locus. All other multiple mutants (*hua2–7 hulk1 hulk2*, *hua2–7 hulk1 hulk3*, *hulk1 hulk2 hulk3* and *hua2–7*/*+ hulk1 hulk2 hulk3*) were derived from a cross between *hua2–7 hulk1* and *hulk2 hulk3* double mutants.

### Artificial microRNAs against *HULK1, HUA2/HULK1* and *HULK2/HULK3* genes

The Web Micro Designer (WMD, http://wmd.weigelworld.org/bin/mirnatools.pl) was used to design amiRNAs against *HULK1*, *HUA2*/*HULK1* and *HULK2*/*HULK3* genes (Schwab *et al*., 2006). The amiRNA sequences were introduced into the Arabidopsis miR319a precursor by overlapping PCR using the primers listed in Table S7. The plasmid pRS300 that contains the mir319a precursor was used as a template (Schwab *et al*., 2006). *HULK1* amiRNA and *HUA2*/*HULK1* amiRNA were recombined into the binary vector pGREENIIS_2100 (Invitrogen, http://www.lifetechnologies.com/), and *HULK2*/*HULK3* amiRNA was recombined into the binary vector pGWB2 (Nakagawa *et al*., 2007). These vectors confer resistance to glufosinate and kanamycin, respectively, which were used for selecting transformants using standard methods.

### Analysis of flowering time

Flowering time was analyzed on the basis of the total number of rosette and cauline leaves produced prior to the node of the first flower. The effect of genotype on flowering time was assessed using anova followed by the Tukey–Kramer procedure to correct for multiple testing among comparisons.

### Gene expression analysis

For quantitative RT–PCR, total RNA was extracted using an RNeasy plant mini kit, together with DNase treatment (Qiagen, http://www.qiagen.com/). RNA was reverse-transcribed using a Maxima first-strand cDNA synthesis kit (Thermo Fisher Scientific, http://www.thermofisher.com/) and/or Superscript II reverse transcriptase (Invitrogen). For *HULK1* amiRNA and *HUA2*/*HULK1* amiRNA experiments, Platinum SYBR Green qPCR SuperMix-UDG (Invitrogen) and an Opticon continuous fluorescence detection system (MJ Research, http://www.bio-rad.com/) were used. For characterization of T–DNA lines and *HULK2*/*HULK3* amiRNA experiments, Maxima SYBR Green/ROX qPCR master mix (Thermo Fisher Scientific) and an Agilent Mx3005P quantitative PCR instrument (Agilent Technologies, http://www.agilent.com/) were used. Primer sequences and amplification efficiencies (*E*) are listed in Table S7. Experimental values for three biological replicates, each analysed in triplicate, were normalized against *TUB4* (AT5G44340) (*HULK1* amiRNA and *HUA2*/*HULK1* amiRNA) or *PEX4* (AT5G25760) (for all other analysis) (Czechowski *et al*., 2005). For statistical analysis, normalized relative quantities were log_2_-transformed, and factorial anova was used to assess the significance of the main effect (plant genotype) (Rieu and Powers, 2009). anova was performed, followed by Tukey's honestly significant difference test.

For the analysis of *HULK* gene expression patterns across tissues and developmental stages, we used the expression microarray AtGenExpress dataset (Schmid *et al*., 2005), RNA-Seq read data and *in situ* localization (see Methods S2).

### Nuclear localization of HULK proteins

For nuclear localization studies, *HULK* cDNAs were amplified using Phusion polymerase (NEB, http://www.neb.com) with the primer pairs listed in Table S7. *HUA2* cDNA was cloned into pEarleyGate 103 (Earley *et al*., 2006), and the *HULK* cDNAs were cloned into pBIN19 35S:attR-YFP (Subramanian *et al*., 2006). *Agrobacterium tumefaciens* was infiltrated into *N. benthamiana* leaves as described previously (de Felippes and Weigel, 2010). A plasmid encoding mCherry–NLS (which is targeted to the nucleus and under the control of a constitutive promoter) was used as a positive control. The p19 protein from tomato bushy stunt virus cloned in pBIN61 was used to suppress gene silencing (Voinnet *et al*., 2000). The infiltrated plants were transferred to a greenhouse, and after 2–3 days, the abaxial epidermis was visualized for fluorescence by confocal laser-scanning microscopy using a Leica TCS SP2 confocal microscope (http://www.leica-microsystems.com/).

### Morphological characterization of *HULK* mutants

Seed clearing for embryo analysis was performed as described by Berleth and Jurgens (1993). The seeds were mounted on microscopic slides in chloral hydrate/water/glycerol (8:2:1), and examined with differential interference contrast optics on a Nikon Eclipse E600 microscope (http://www.nikon.com/). Pollen viability tests were performed as described by Alexander (1969). Anthers were visualized under differential interference contrast optics on a Nikon Eclipse E600 microscope. Flower samples for scanning electron microscopy were fixed for 5 min in 100% methanol, and were rinsed with 100% ethanol five times. Samples were examined using a Hitachi S800 electron microscope (http://www.hitachi.com/).

### Analysis of *HULK* gene expression patterns and expression profiling of *HULK* mutants

Analysis of *HULK* gene expression pattern by means of RNA-Seq, *in situ* hybridization and GUS staining are described in Methods S2 (Weigel and Glazebrook, 2002; Kover *et al*., 2009; Gan *et al*., 2011; Trapnell *et al*., 2012).

Transcriptome profiling using Illumina RNA-Seq (http://www.illumina.com/) with biological replication for seedlings of Col–0, *hua2–7* single mutants, *hua2–7 hulk1* double mutants and *hua2–7 hulk1 hulk2* triple mutants is described in Methods S3 (Anders and Huber, 2010; Doherty and Kay, 2010; Du *et al*., 2010; Gan *et al*., 2011; Trapnell *et al*., 2012).

## Supporting Information

**Figure S1.** Protein alignment, conservation, consensus sequence and percentage similarity, and identity matrix of domains found in the Arabidopsis HULK proteins.

**Figure S2.** Rooted phylogram of HULK-like proteins.

**Figure S3.** Gene expression levels of *HUA2*, *HULK1*, *HULK2* and *HULK3* in various tissues and organs.

**Figure S4.** *HULK2* and *HULK3* gene expression during embryo and pollen development as detected by *in situ* hybridization and GUS staining.

**Figure S5.** *HUA2* and *HULK1–3* mutant alleles used in this study.

**Figure S6.** Alignments of Illumina RNA-Seq reads to the *HUA2*, *HULK1* and *HULK2* loci in the *hua2–7*, *hulk1* and *hulk2* genetic backgrounds.

**Figure S7.** Characterization of T_2_ *HULK1* amiRNA and *HUA2*/*HULK1* amiRNA plants.

**Figure S8.** Quantitative RT–PCR analysis of the effect of amiRNA targeting *HULK2*/*HULK3*.

**Figure S9.** Log_2_-transformed RNA-Seq gene expression levels.

**Figure S10.** Venn diagram of the top 20 GO functional categories in the *hua2–7*, *hua2–7 hulk1* and *hua2–7 hulk1 hulk2* mutants.

**Table S1.** Number of RNA-Seq reads aligned per sample by genotype and biological replicate.

**Table S2.** Gene expression per TAIR10 gene model inferred from RNA-Seq data for Col–0 and *HULK* mutant plants.

**Table S3.** Differentially expressed genes identified by RNA-Seq in *hua2–7*, *hua2–7 hullk1* and *hua2–7 hulk1 hulk2* relative to Col–0.

**Table S4.** Significantly represented (*P *<* *0.05) GO slim terms in sets of differentially expressed genes in *hua2–7*, *hua2–7 hullk1* and *hua2–7 hulk1 hulk2* plants relative to Col–0.

**Table S5.** Loss-of-function phenotypes of plants bearing mutations in differentially expressed genes identified by RNA-Seq in single, double and triple mutants.

**Table S6.** List of putative HULK-like proteins used in this study.

**Table S7.** List of primers used in this study.

**Methods S1.** Preparation of dataset for phylogenetic analysis of *HUA2-LIKE* sequences in Embryophytes.

**Methods S2.** Analysis of *HULK* gene expression patterns.

**Methods S3.** Expression profiling of *HULK* mutants.
