# Supplementary material for: A plant-specific HUA2-LIKE (HULK) gene family in Arabidopsis thaliana is essential for development
Source: Plant J. 2014 Aug 28;80(2):242–54. doi: 10.1111/tpj.12629 (PMC4283595; doi:10.1111/tpj.12629)
Supplement: Supplementary file 1 — Figure S1. Protein alignment, conservation, consensus sequence and percentage similarity, and identity matrix of domains found in the Arabidopsis HULK proteins. [file tpj0080-0242-sd1.pdf]

a

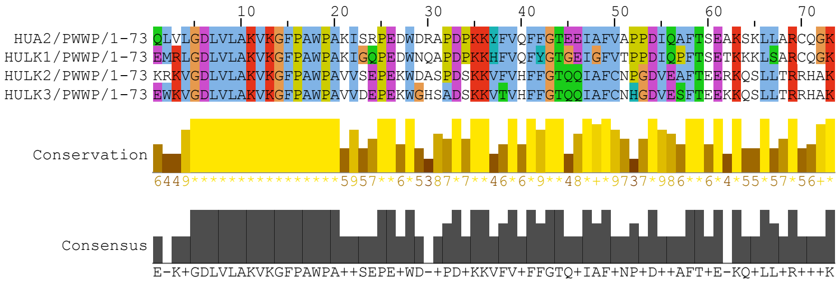

	HUA2	HULK1	HULK2	HULK3
HUA2		83.6	71.2	65.8
HULK1	78.1		68.5	64.4
HULK2	61.6	53.4		87.7
HULK3	53.4	50.7	86.3	

Sequence similarity, %

Sequence identity, %

b

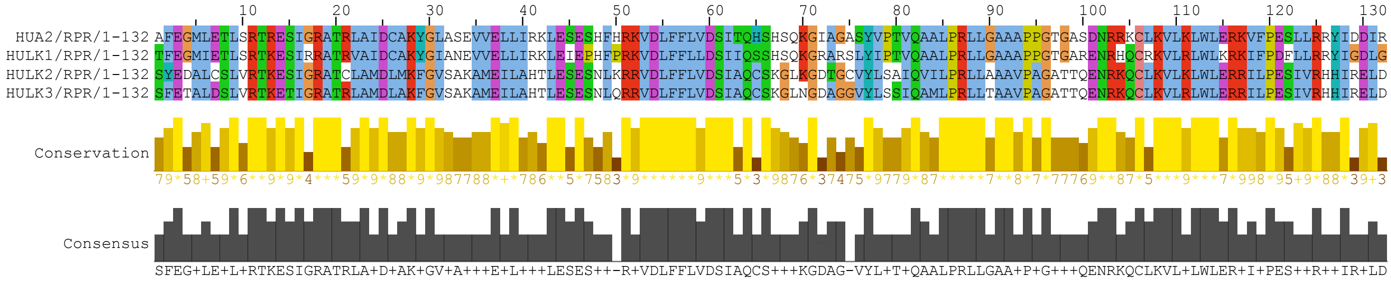

	HUA2	HULK1	HULK2	HULK3
HUA2		85.6	72.7	74.2
HULK1	76.5		69.7	71.2
HULK2	52.3	47.0		91.7
HULK3	53.8	50.0	88.6	

Sequence similarity, %

Sequence identity, %

Figure S1. Protein alignment, conservation, consensus sequence and % similarity and identity matrix of the PWWP (**a**) and RPR (**b**) domains found in the Arabidopsis HULK proteins.
