# Supplementary material for: A plant-specific HUA2-LIKE (HULK) gene family in Arabidopsis thaliana is essential for development
Source: Plant J. 2014 Aug 28;80(2):242–54. doi: 10.1111/tpj.12629 (PMC4283595; doi:10.1111/tpj.12629)
Supplement: Supplementary file 2 — Figure S2. Rooted phylogram of HULK-like proteins. [file tpj0080-0242-sd2.pdf]

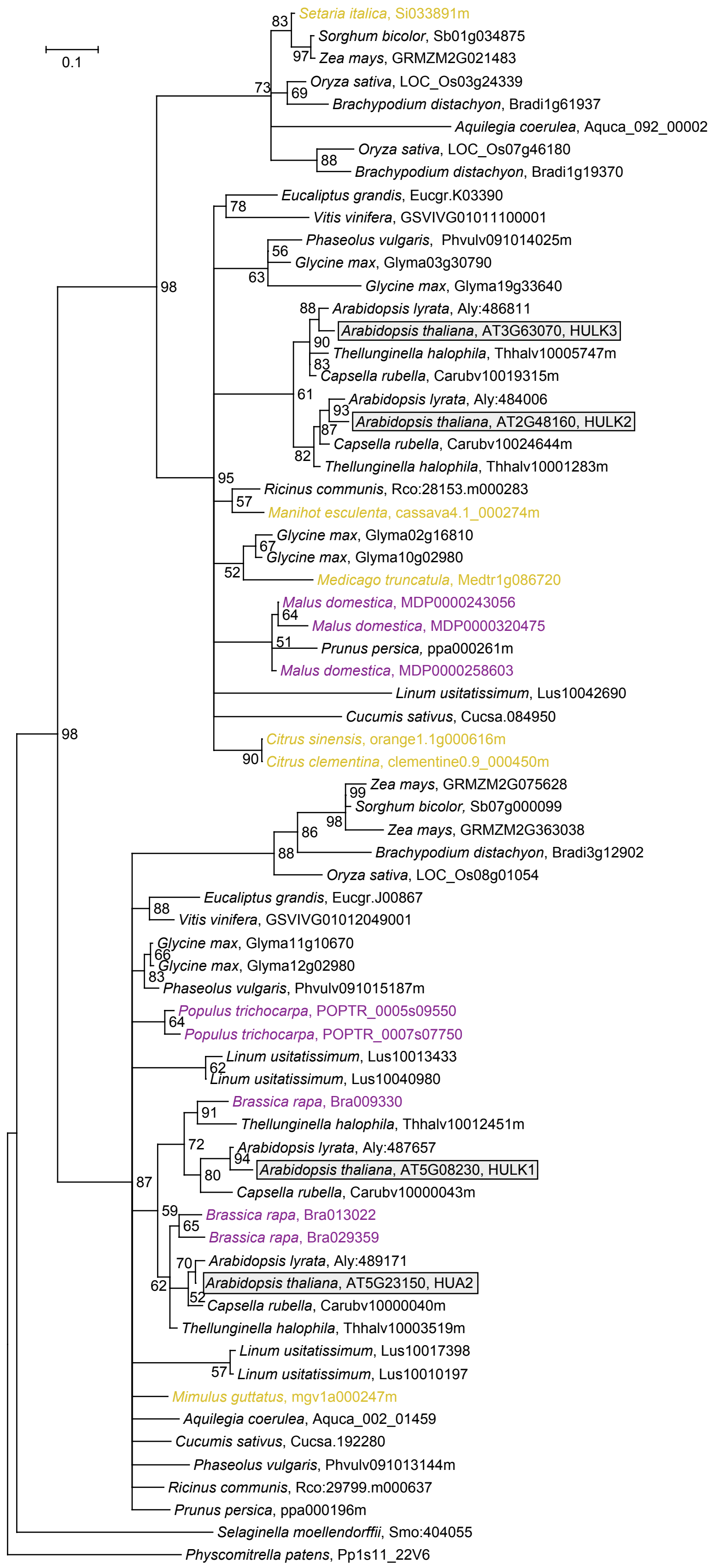

Figure S2. Rooted phylogram of amino acid sequences in domains of 69 HULK-like proteins of 28 Embryophyte species with *Physcomitrella patens* as an outgroup based on maximum likelihood. Sequences highlighted in purple: multiple HUA2-like proteins from species having representatives of only the HUA2/HULK1 or the HULK2/HULK3 clades; sequences highlighted in yellow: species represented by a single HUA2-like protein. The scale bar is a number of amino acid substitutions per site. Support values are puzzle support values.
