# Supplementary material for: A plant-specific HUA2-LIKE (HULK) gene family in Arabidopsis thaliana is essential for development
Source: Plant J. 2014 Aug 28;80(2):242–54. doi: 10.1111/tpj.12629 (PMC4283595; doi:10.1111/tpj.12629)
Supplement: Supplementary file 3 — Figure S3. Gene expression levels of HUA2, HULK1, HULK2 and HULK3 in various tissues and organs. [file tpj0080-0242-sd3.pdf]

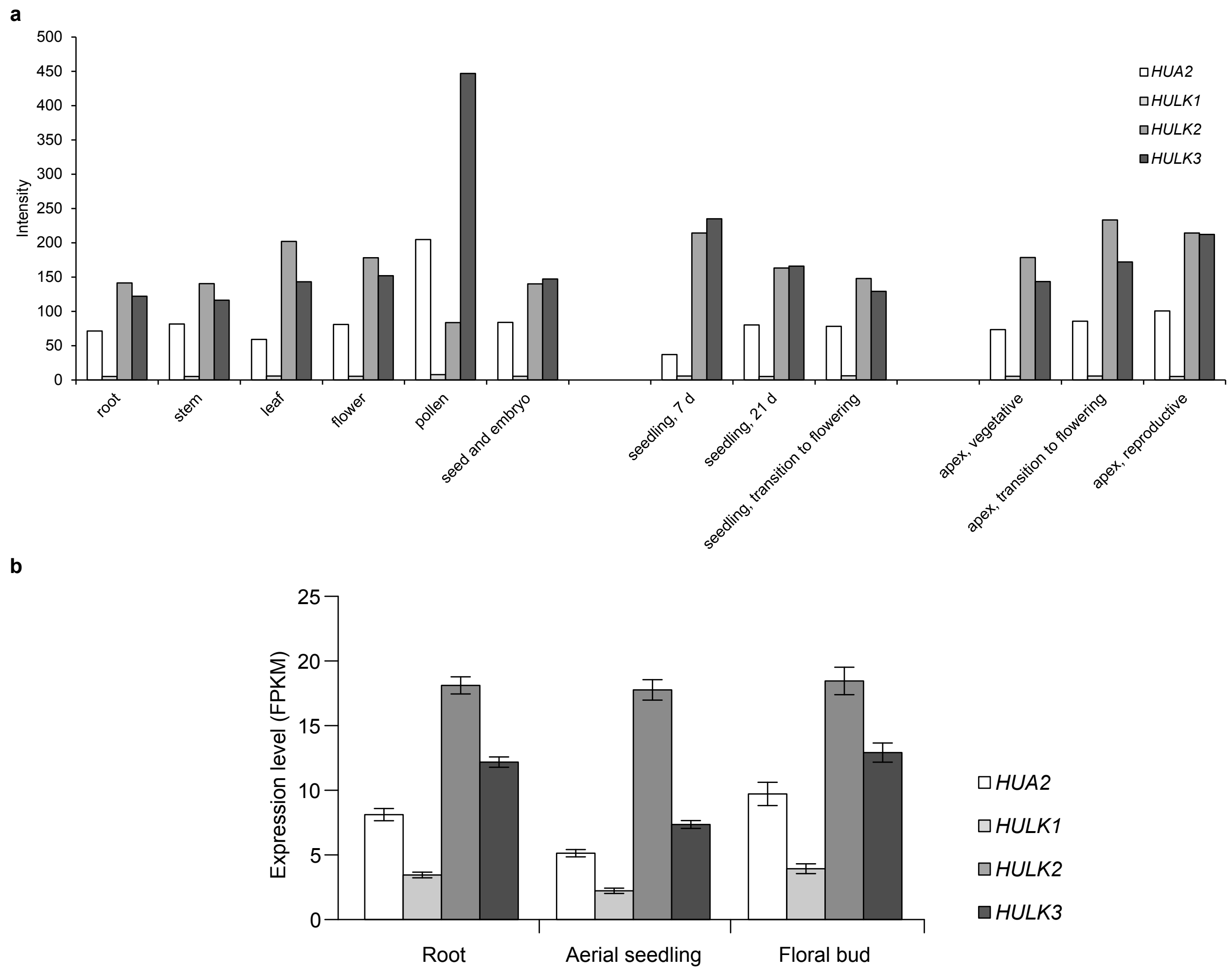

Figure S3. Gene expression levels of *HUA2*, *HULK1*, *HULK2* and *HULK3* in various tissues and organs. (**a**) Data obtained from the developmental set of AtGenExpress for accession Col-0 (http://jsp.weigelworld.org/expviz/expviz.jsp, Schmid *et al.*, 2005). (**b**) Mean levels of expression for each *HULK* gene as averaged from RNA-Seq data over each MAGIC founder accession for each of three tissues (*n* = 19 for each tissue; whole root of 10-day-old seedlings, aerial seedlings at emergence of the fourth true leaf, and stage 12 floral buds as indicated from left to right). FPKM is Fragments Per Kilobase of transcript per Million mapped reads. Error bars denote ± 2 standard errors of the mean.
