# Supplementary material for: A plant-specific HUA2-LIKE (HULK) gene family in Arabidopsis thaliana is essential for development
Source: Plant J. 2014 Aug 28;80(2):242–54. doi: 10.1111/tpj.12629 (PMC4283595; doi:10.1111/tpj.12629)
Supplement: Supplementary file 4 — Figure S4. HULK2 and HULK3 gene expression during embryo and pollen development as detected by in situ hybridization and GUS staining. [file tpj0080-0242-sd4.pdf]

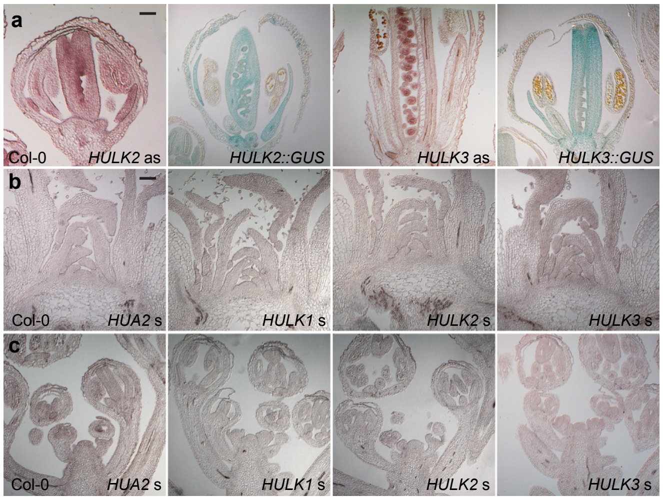

Figure S4. *HULK2* and *HULK3* gene expression in embryo and pollen development as detected by *in situ* hybridization and GUS staining (**a**). Negative control for *HULK* gene expression in vegetative shoot and inflorescence apices as detected by *in situ* hybridization. Longitudinal sections of vegetative shoot (**b**) and inflorescence apices (**c**) hybridized with sense (s) probes to the four *HULK* genes as indicated. All sections are from wildtype (Col-0) plants. Scale bars: 35 µm in a, 100 µm in b and c.
