# Supplementary material for: A plant-specific HUA2-LIKE (HULK) gene family in Arabidopsis thaliana is essential for development
Source: Plant J. 2014 Aug 28;80(2):242–54. doi: 10.1111/tpj.12629 (PMC4283595; doi:10.1111/tpj.12629)
Supplement: Supplementary file 5 — Figure S5. HUA2 and HULK1–3 mutant alleles used in this study. [file tpj0080-0242-sd5.pdf]

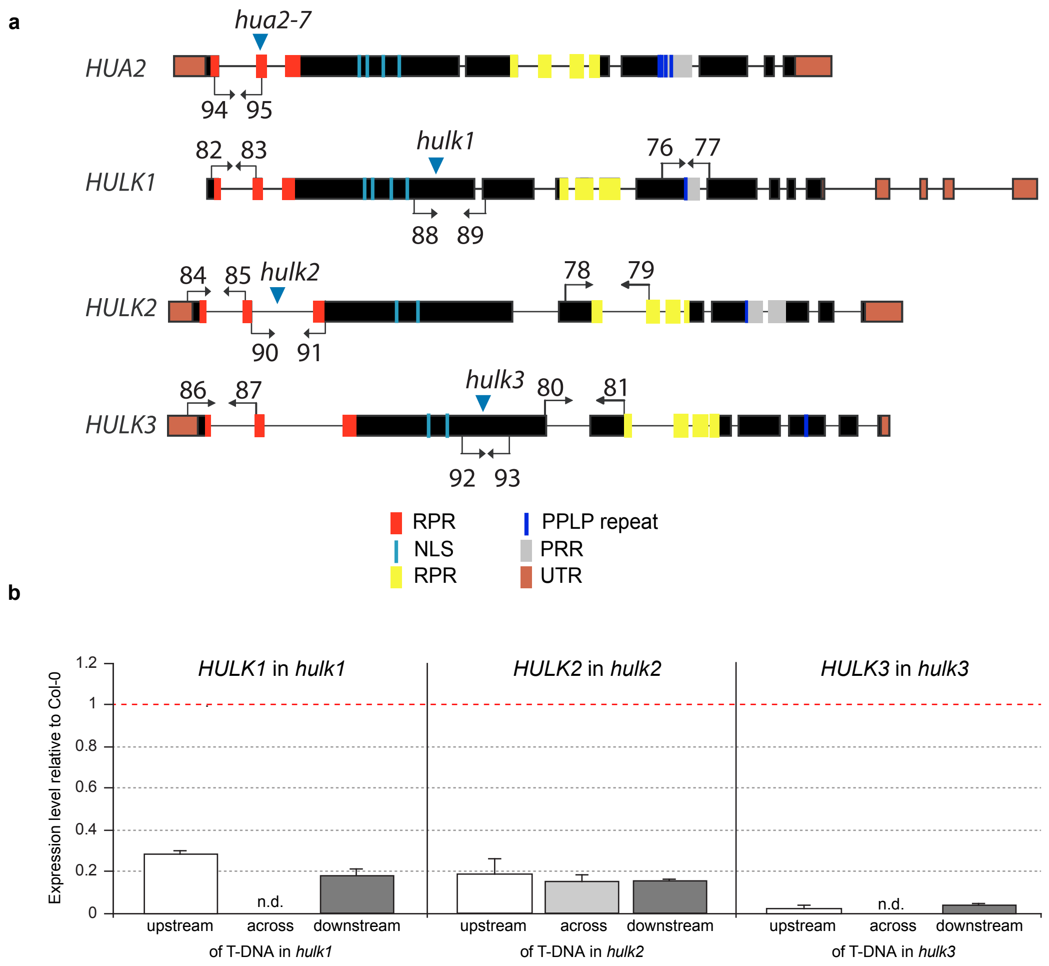

Figure S5. *HUA2* and *HULK1-3* mutant alleles used in this study. (**a**) Position of T-DNA insertions in the *hua2-7*, *hulk1-1*, *hulk2-1* and *hulk3-1* alleles employed in this study. At each locus, arrows indicate the location and direction of primers used to assay expression by RT-qPCR. Exons are indicated by rectangles, introns by lines. (**b**) Mean ± standard error of the mean fold changes of *HULK1*, *HULK2* and *HULK3* relative to Col-0 in T-DNA insertion lines as assessed by RT-qPCR ($n = 3$). n.d., not detected.
