# Supplementary material for: A plant-specific HUA2-LIKE (HULK) gene family in Arabidopsis thaliana is essential for development
Source: Plant J. 2014 Aug 28;80(2):242–54. doi: 10.1111/tpj.12629 (PMC4283595; doi:10.1111/tpj.12629)
Supplement: Supplementary file 6 — Figure S6. Alignments of Illumina RNA-Seq reads to the HUA2, HULK1 and HULK2 loci in the hua2–7, hulk1 and hulk2 genetic backgrounds. [file tpj0080-0242-sd6.pdf]

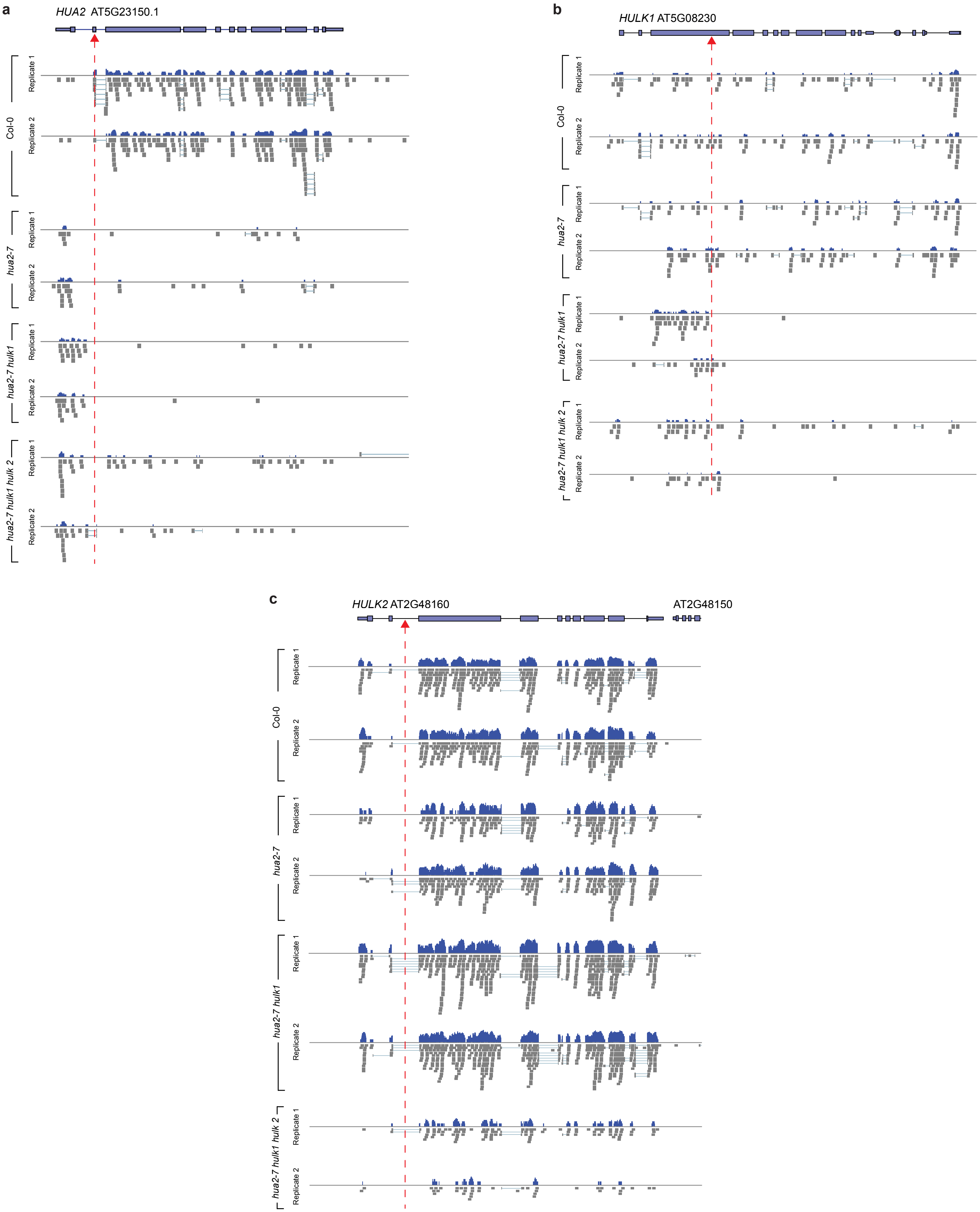

Figure S6. Alignments of Illumina RNA-Seq reads to the *HUA2*, *HULK1* and *HULK2* loci in the *hua2-7*, *hulk1* and *hulk2* genetic backgrounds as indicated. Gene models are as shown at top (thick bars indicate coding sequences), and red arrows indicate positions of T-DNA insertions. Light blue lines indicate read alignments that span exons junctions. All aligned reads for a given genotype are shown. Normalized expression values (reads per million mapped, or RPM) for each *HULK* gene are given in Table S2.
