# Supplementary material for: A plant-specific HUA2-LIKE (HULK) gene family in Arabidopsis thaliana is essential for development
Source: Plant J. 2014 Aug 28;80(2):242–54. doi: 10.1111/tpj.12629 (PMC4283595; doi:10.1111/tpj.12629)
Supplement: Supplementary file 7 — Figure S7. Characterization of T2 HULK1 amiRNA and HUA2/HULK1 amiRNA plants. [file tpj0080-0242-sd7.pdf]

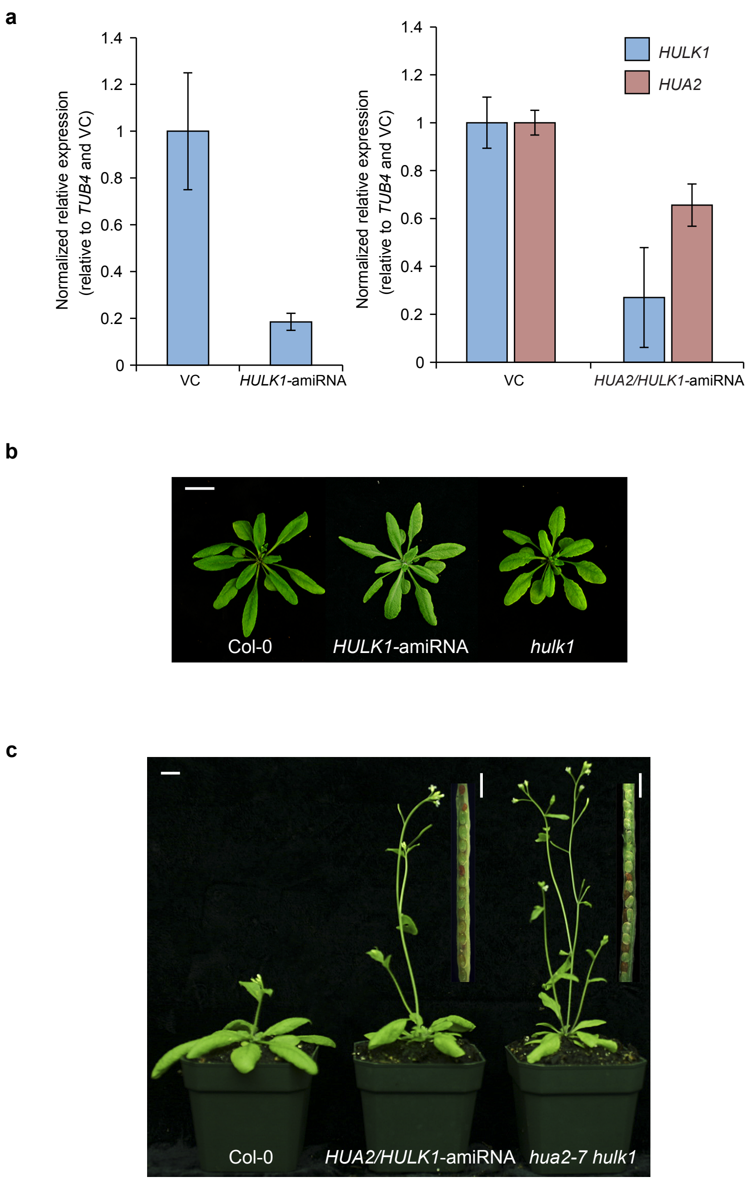

Figure S7. Characterization of T2 *HULK1*-amiRNA and *HUA2/HULK1*-amiRNA plants. (**a**) Expression levels of *HULK1* and *HUA2* in 7-day old seedlings and leaves as determined by RT-qPCR. Values represent means of two replicates ± standard error of the mean. (**b**) Phenotypes of Col-0, *HULK1*-amiRNA and *hulk1* plants. (**c**) Plant and silique phenotypes in Col-0, *HUA2/HULK1*-amiRNA and *hua2-7 hulk1* lines. VC – plants transformed with empty vector. Scale bars: 1 cm in b and c (main figure), 1 mm in c (silique inset).
