# Supplementary material for: A plant-specific HUA2-LIKE (HULK) gene family in Arabidopsis thaliana is essential for development
Source: Plant J. 2014 Aug 28;80(2):242–54. doi: 10.1111/tpj.12629 (PMC4283595; doi:10.1111/tpj.12629)
Supplement: Supplementary file 8 — Figure S8. Quantitative RT–PCR analysis of the effect of amiRNA targeting HULK2/HULK3. [file tpj0080-0242-sd8.pdf]

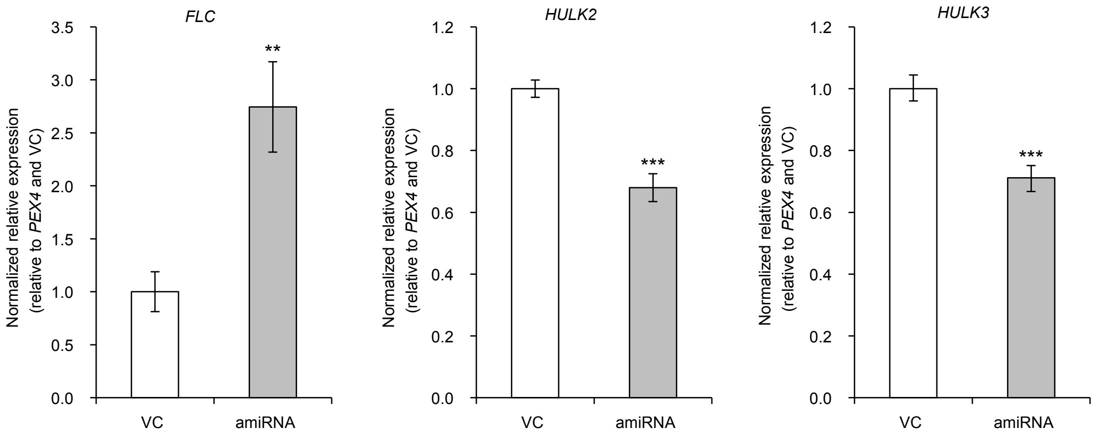

Figure S8. RT-qPCR analysis of the effect of amiRNA targeting *HULK2/HULK3* on levels of *FLC, HULK2* and *HULK3* in T1 Col-0 seedlings transformed with either *HULK2/HULK3*-amiRNA construct (labelled amiRNA, $n = 20$) or empty vector (labelled VC, $n = 12$). Mean ± standard error of the mean fold changes of *FLC*, *HULK2* and *HULK3*. Asterisks indicate significant differences in expression levels between treatments (Tukey HSD test, ** - $P < 0.01$, *** - $P < 0.001$).
