# Supplementary material for: A plant-specific HUA2-LIKE (HULK) gene family in Arabidopsis thaliana is essential for development
Source: Plant J. 2014 Aug 28;80(2):242–54. doi: 10.1111/tpj.12629 (PMC4283595; doi:10.1111/tpj.12629)
Supplement: Supplementary file 9 — Figure S9. Log2-transformed RNA-Seq gene expression levels. [file tpj0080-0242-sd9.pdf]

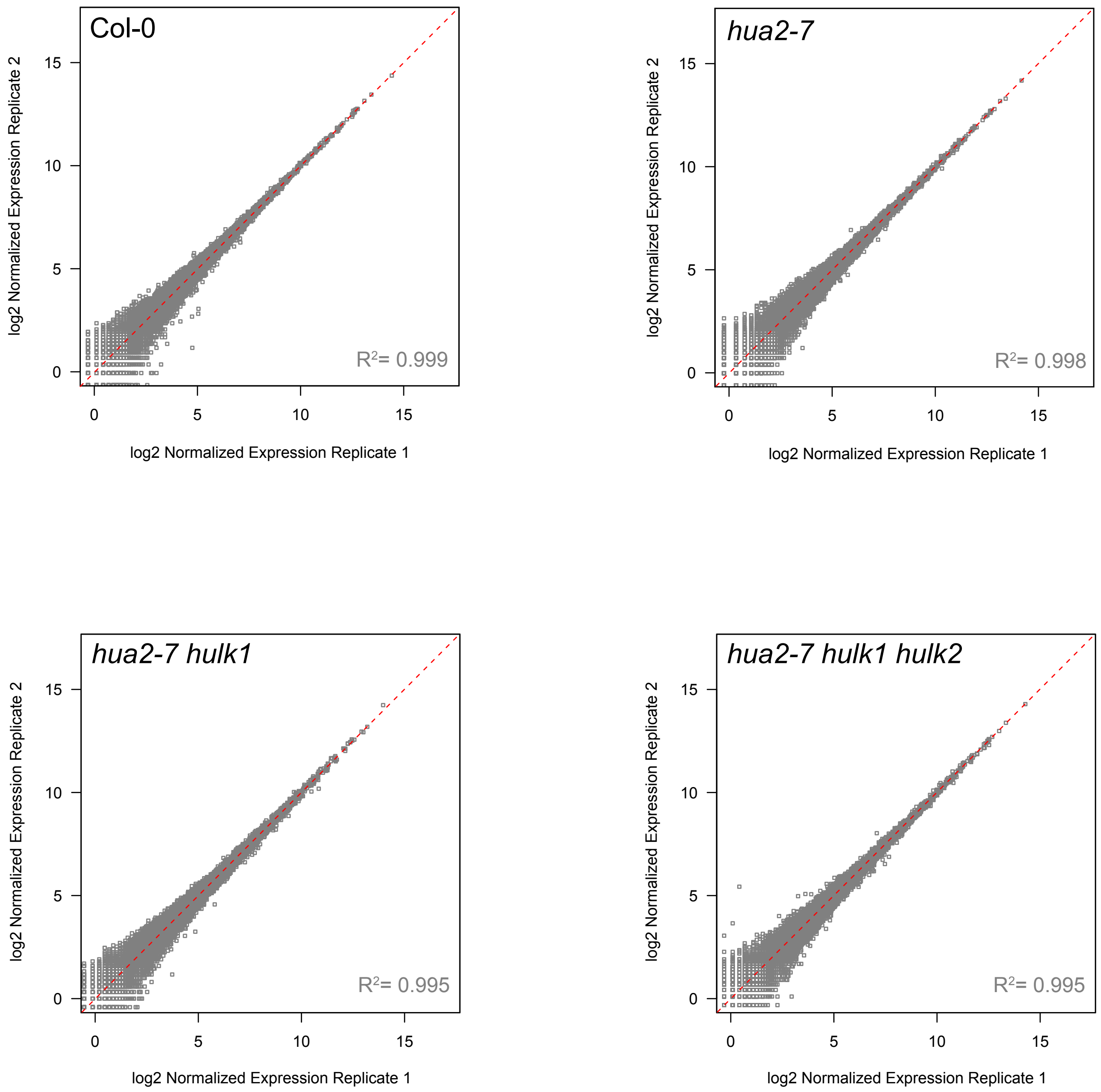

Figure S9. Log_2 transformed RNA-Seq gene expression levels (reads per million mapped) of biologically replicated seedling samples with genotypes as indicated. R^2 values were calculated with all genes for which five or more reads mapped in at least one biological replicate.
