# Supplementary material for: A plant-specific HUA2-LIKE (HULK) gene family in Arabidopsis thaliana is essential for development
Source: Plant J. 2014 Aug 28;80(2):242–54. doi: 10.1111/tpj.12629 (PMC4283595; doi:10.1111/tpj.12629)
Supplement: Supplementary file 10 — Figure S10. Venn diagram of the top 20 GO functional categories in the hua2–7, hua2–7 hulk1 and hua2–7 hulk1 hulk2 mutants. [file tpj0080-0242-sd10.pdf]

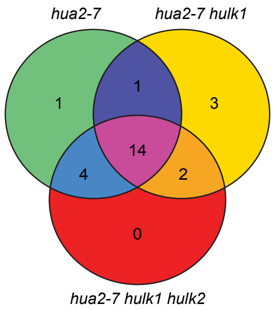

Common categories in *hua2-7*, *hua2-7 hulk1* and *hua2-7 hulk1 hulk2*:

GO:0050896 response to stimulus
GO:0009607 response to biotic stimulus
GO:0051704 multi-organism process
GO:0006950 response to stress
GO:0009719 response to endogenous stimulus
GO:0009605 response to external stimulus
GO:0009628 response to abiotic stimulus
GO:0009791 post-embryonic development
GO:0050789 regulation of biological process
GO:0050794 regulation of cellular process
GO:0007165 signal transduction
GO:0019222 regulation of metabolic process
GO:0006350 transcription
GO:0010468 regulation of gene expression

Common categories in *hua2-7* and *hua2-7 hulk1*:

GO:0065007 biological regulation

Common categories in *hua2-7* and *hua2-7 hulk1 hulk2*:

GO:0048608 reproductive structure development
GO:0003006 reproductive developmental process
GO:0022414 reproductive process
GO:0000003 reproduction

Common categories in *hua2-7 hulk1* and *hua2-7 hulk1 hulk2*:

GO:0060255 regulation of macromolecule metabolic process
GO:0009908 flower development

Categories only in *hua2-7*:

GO:0032501 multicellular organismal process

Categories only in *hua2-7 hulk1*:

GO:0040007 growth
GO:0006629 lipid metabolic process
GO:0006139 nucleobase, nucleoside, nucleotide and nucleic acid metabolic process

Categories only in *hua2-7 hulk1 hulk2*:

none

Figure S10. Venn diagram (above) comparing and contrasting the top 20 GO functional categories (below) in the *hua2-7*, *hua2-7 hulk1* and *hua2-7 hulk1 hulk2* mutants. Genes used for the analysis were detected as differentially expressed by RNA-Seq profiling.
