# Supplementary material for: A plant-specific HUA2-LIKE (HULK) gene family in Arabidopsis thaliana is essential for development
Source: Plant J. 2014 Aug 28;80(2):242–54. doi: 10.1111/tpj.12629 (PMC4283595; doi:10.1111/tpj.12629)
Supplement: Supplementary file 11 — Table S1. Number of RNA-Seq reads aligned per sample by genotype and biological replicate. [file tpj0080-0242-sd11.doc]

**Table S1**. Total number of reads mapped per sample.

|  | **Col-0** | |  | ***hua2-7*** | |  | ***hua2-7 hulk1*** | |  | ***hua2-7 hulk1 hulk2*** | |
| --- | --- | --- | --- | --- | --- | --- | --- | --- | --- | --- | --- |
|  | Replicate 1 | Replicate 2 |  | Replicate 1 | Replicate 2 |  | Replicate 1 | Replicate 2 |  | Replicate 1 | Replicate 2 |
| **Total reads mapped** | 3,711,654 | 3,126,286 |  | 2,367,426 | 3,037,877 |  | 4,355,883 | 3,999,070 |  | 3,745,621 | 3,730,250 |
