# Supplementary material for: A plant-specific HUA2-LIKE (HULK) gene family in Arabidopsis thaliana is essential for development
Source: Plant J. 2014 Aug 28;80(2):242–54. doi: 10.1111/tpj.12629 (PMC4283595; doi:10.1111/tpj.12629)
Supplement: Supplementary file 18 — Methods S1. Preparation of dataset for phylogenetic analysis of HUA2-LIKE sequences in Embryophytes. [file tpj0080-0242-sd18.docx]

**Methods S1: Preparation of dataset for phylogenetic analysis of** *HUA2-LIKE* **sequences in Embryophytes**

We identified genes with homology to *HUA2* in the *Arabidopsisthaliana* (Arabidopsis) genome by performing BLAST searches with *HUA2* nucleotide and protein sequences against annotated genes (TAIR8 annotation release). Subsequently, theHUA2 protein sequence from Arabidopsiswas used to perform a BLASTP search against Embryophyte gene families in the Phytozome v. 8 database using the following parameters: E = -1, substitution matrix – BLOSUM62, W = 3, gaps – allowed, low-complexity filter – on and singletons exclusion ([Goodstein *et al.*, 2012](#_ENREF_7)). A single family containing 84 members of Tudor/PWWP/MBT domain-containing proteins was retrieved (Supplemental Table S7). Sequences were aligned using MUSCLE v.3.8.31 with the default parameters ([Edgar, 2004](#_ENREF_5)).Alignment was manually curated to retain full-length, high quality sequences. We retained 69 sequences that were re-aligned using MUSCLE and alignment was further processed using Gblocks v.0.91b with default stringency ([Castresana, 2000](#_ENREF_2)). The final blocks alignment contained 179 AA positions corresponding to the PWWP and RPR domains.
