# Supplementary material for: A plant-specific HUA2-LIKE (HULK) gene family in Arabidopsis thaliana is essential for development
Source: Plant J. 2014 Aug 28;80(2):242–54. doi: 10.1111/tpj.12629 (PMC4283595; doi:10.1111/tpj.12629)
Supplement: Supplementary file 19 — Methods S2. Analysis of HULK gene expression patterns. [file tpj0080-0242-sd19.docx]

**Methods S2: Analysis of *HULK* gene expression patterns**

*RNA-seq*

We used RNA-seq read data to assess the relative expression level of each *HULK* gene in roots, aerial seedlings and stage 12 floral bud tissues in each of 19 accessions that are parents of the Multiparent Advanced Generation InterCross genetic mapping resource ([Kover *et al.*, 2009](#_ENREF_8)). Briefly, RNA-seq reads for two of these accessions (Col-0 and Can-0) were previously published (Gene Expression Omnibus series number GSE30795), and analogous data for the remaining 17 accessions is released as part of this study (for methods, see ([Gan *et al.*, 2011](#_ENREF_6))). For each of the 57 accession and tissue combinations, RNA-seq reads were aligned to the Arabidopsis genome and TAIR10 annotation with TopHat 2.0.9 and Bowtie2 2.1.0 (parameters used for mapping were: -a 5 -i 5 -I 32000 --b2-very-sensitive --segment-mismatches 2), and Cufflinks 2.1.1 was used to calculate normalized gene expression levels using default parameters ([Trapnell *et al.*, 2012](#_ENREF_9)).

*In situ hybridization*

Additionally, *in situ* hybridization with gene-specific probes was used to assess spatial expression patterns. *HUA2* and *HULK1* specific cDNA probes were amplified using primers listed in Supplemental Table S6. The PCR products were cloned into the pGEM-T easy vector. To obtain the *HULK2* probe, a SpeI and PstI cDNA fragment was cloned into the pGEM-T easy vector. A *HULK3* specific PstI cDNA fragment was cloned in pGEM-T easy vector. Oligo-nucleotide primer sequences for PCR amplification of full length *HULK1, HULK2* and *HULK3* are listed in Supplemental Table S6. Finally, the RNA *in situ* hybridization was performed with *HULK* gene probes containing nucleotides 461-1357 relative to start codon for *HUA2*, 508-1122 for *HULK1*, 1675-2301 for *HULK2* and 1400-2046 for *HULK3*. Vegetative apices were collected from 28-days-old short day grown plants and inflorescence apices were collected from long day grown flowering plants. Non-radioactive RNA *in situ* hybridization was performed as previously described ([Weigel and Glazebrook, 2002](#_ENREF_10)).

*GUS staining*

For *HULK2::GUS,* 876 bp fragment of *HULK2* promoter was amplified with Expand High Fidelity Enzyme (Roche). The PCR product was cloned into pGEM-T Easy vector for sequence verification. The pGEM-T easy vector was digested with *EcoRI* enzyme. The digested product was gel purified and cloned in to the pRITA I vector. The pRITA I vector containing the *HULK2* promoter, GUS sequence and NOS terminator were excised with *NotI* and ligated into the pMLBart binary vector. The orientation of the insert was determined by restriction digestion with *XhoI.*

For *HULK3::GUS,* a 1203 bp fragment of *HULK3* promoter was amplified with Expand High Fidelity Enzyme (Roche). The PCR product was cloned into pGEM-T Easy vector for sequence verification. The pGEM-T easy vector was digested with *EcoRI* enzyme. The digested product was gel purified and cloned in to the pRITA I vector. The orientation of the insert in pRITA I was confirmed with *XhoI* digestion. The pRITA I vector containing the *HULK2* promoter, GUS sequence and NOS terminator were excised with *NotI* and ligated into pMLBart binary vector. The orientation of the insert was determined by restriction digestion with *HindIII.*

GUS staining was performed as previously described ([Weigel and Glazebrook, 2002](#_ENREF_10)). Primers used in cloning the *HULK* promoters are indicated in Supplemental Table S6.
