# Supplementary material for: A plant-specific HUA2-LIKE (HULK) gene family in Arabidopsis thaliana is essential for development
Source: Plant J. 2014 Aug 28;80(2):242–54. doi: 10.1111/tpj.12629 (PMC4283595; doi:10.1111/tpj.12629)
Supplement: Supplementary file 20 — Methods S3. Expression profiling of HULK mutants. [file tpj0080-0242-sd20.docx]

**Methods S3: Expression profiling of *HULK* mutants**

*RNA collection and preparation of mRNA-seq libraries for HULK mutants*

Col-0, *hua2-7*, *hua2-7 hulk1*, and *hua2-7 hulk1 hulk2* seedling tissue, with biological replication, was collected for the production of Illumina mRNA sequencing (RNA-seq) libraries. Seeds were sterilized with chlorine gas for 3 hours, and stratified for 5 days in 0.1% agarose at 4°C. Plants were germinated and grown on Sunshine Mix 4 Aggregate Plus soil (cat. no. LA4, Sun Gro Horticulture, Bellevue, WA, USA) supplemented with ~0.03 g of Miracle-Grow® all-purpose plant food in 10 cm square pots in a Percival AR-66L environment growth chamber (see Gan et al., 2011 for growth details). All genotypes were grown at 20°C under long day growth conditions (16:8 hours light:dark). Approximately 20 seedlings were grown per pot, and 9 pots were planted per genotype. Plants were rotated through the chamber daily to eliminate potential environmental effects on development resulting from position within the growth chamber. To secure developmental uniformity of samples, the seedling tissues were collected when the 4th true leaf was visible. The aerial portions of seedlings were harvested by detachment just below the cotyledons. Twenty seedlings were collected and combined for each genotype and biological replicate. To minimize environmental effects, seedlings for a given replicate were collected in approximately equal numbers from each pot within the growth chamber for a given genotype. Biological replicates for each genotype were collected simultaneously. To eliminate circadian-dependent effects on gene expression (i.e., see ([Doherty and Kay, 2010](#_ENREF_3))), tissue collections were carried out at 8 ± 0.5 hours into the light cycle as described by ([Gan *et al.*, 2011](#_ENREF_6)). Tissue was collected directly into liquid nitrogen and subsequently stored at -80°C. Total RNA isolation and DNase treatment was performed as described previously ([Gan *et al.*, 2011](#_ENREF_6)). Messenger RNA (mRNA) was purified from 35 µg of total RNA using two rounds of selection with DynalBeads® Oligo (dT)_25_ beads (cat. no. 610.05, Invitrogen). Isolated mRNA was suspended in RNase-free water. To facilitate analysis of multiple RNA samples, with replication, mRNA was used to construct barcoded RNA-seq libraries with methods adapted from the Illumina mRNA sample preparation protocol (cat. no 1004894 Rev.A; ([Gan *et al.*, 2011](#_ENREF_6))). The concentration and size distribution of each library was evaluated on a Bioanalyzer 2100 with DNA 1000 Kits (Agilent, cat. no. 5067-1504); in all cases, the observed library sizes agreed closely with that expected from the size selection. Template concentrations were adjusted to 10 nM with a solution of 10 mM Tris-HCl, pH 8.5, containing 0.1% (v/v) Tween 20.

*Generation and quantification of RNA-seq reads for HULK mutants*

Illumina single-read cluster generation and sequencing was performed according to manufacturer’s instructions. Briefly, flow-cell preparation on a Cluster Station was performed with v5 Illumina Single Read Cluster Generation Kits (cat. no. GD-203-5001) using the “SR_Amplification_Linearization_Blocking_PrimerHyb_v7” Cluster Station workflow. For cluster generation, equal volumes of four barcoded 10 nM libraries were combined and diluted to 8 pM for flow-cell hybridization. All sequencing was performed on a Genome Analyzer IIx (GAIIx) instrument using v5 Sequencing Kits (cat. no. 15003925) and with an Illumina supported modification of the “GA2_76Cycle_SR_v7.xml” sequencing program to perform 82 cycles of imaging. We used version SCS2.6 of the GAIIx control software with Real Time Analysis (RTA) enabled and set to use “per lane” parameters for base-calling. RNA-seq reads were processed, quality filtered, trimmed and assigned to specific libraries using barcode sequences as described by ([Gan *et al.*, 2011](#_ENREF_6)) (resultant reads were 78 bp in length following barcode removal).

*Detection and analysis of differentially expressed genes from aligned RNA-seq reads*

RNA-seq read alignments to gene models and expression quantification were performed using the Bowtie/Tophat software package ([Trapnell *et al.*, 2012](#_ENREF_9)) and Python scripts. The following parameters were used for performing read alignment: -a 5, -i 5, -I 32000, --solexa1.3-quals, -g 1, --segment-mismatches 3, -p 5 with the TAIR10 annotation. To assess differential gene expression, we used the DESeq R package ([Anders and Huber, 2010](#_ENREF_1)). Raw read count data from alignments was used as input for DESeq and pair-wise comparisons were examined between Col-0 and each mutant genotype assayed (three comparisons in total: Col-0 vs. *hua2-7*, Col-0 vs. *hua2-7 hulk1,* and Col-0 vs. *hua2-7 hulk1 hulk2*). Genes with a FDR adjusted *P*-value < 0.05 by the Benjamini-Hochberg procedure, as implemented in DESeq, and greater than a 2-fold change were defined as differentially expressed for downstream analyses (Table S3). Gene Ontology enrichment analysis was carried out using the online GO analysis toolkit agriGO (<http://bioinfo.cau.edu.cn/agriGO/index.php>, ([Du *et al.*, 2010](#_ENREF_4))). Plant GOslim terms enriched in our gene set were identified using a Fisher exact test with significance 0.05 (Supplemental Table S4).
