# Supplementary material for: A plant-specific HUA2-LIKE (HULK) gene family in Arabidopsis thaliana is essential for development
Source: Plant J. 2014 Aug 28;80(2):242–54. doi: 10.1111/tpj.12629 (PMC4283595; doi:10.1111/tpj.12629)
Supplement: Supplementary file 21 [file tpj0080-0242-sd21.docx]

SUPPORTING EXPERIMENTAL PROCEDURES

**Methods S1: Preparation of dataset for phylogenetic analysis of HUA2-like sequences in Embryophytes**

We identified genes with homology to *HUA2* in the *Arabidopsis* *thaliana* (Arabidopsis) genome by performing BLAST searches with *HUA2* nucleotide and protein sequences against annotated genes (TAIR8 annotation release). Subsequently, the HUA2 protein sequence from Arabidopsis was used to perform a BLASTP search against Embryophyte gene families in the Phytozome v. 8 database using the following parameters: E = -1, substitution matrix – BLOSUM62, W = 3, gaps – allowed, low-complexity filter – on and singletons exclusion ([Goodstein *et al.*, 2012](#_ENREF_7)). A single family containing 84 members of Tudor/PWWP/MBT domain-containing proteins was retrieved (Supplemental Table S7). Sequences were aligned using MUSCLE v.3.8.31 with the default parameters ([Edgar, 2004](#_ENREF_5)). Alignment was manually curated to retain full-length, high quality sequences. We retained 69 sequences that were re-aligned using MUSCLE and alignment was further processed using Gblocks v.0.91b with default stringency ([Castresana, 2000](#_ENREF_2)). The final blocks alignment contained 179 AA positions corresponding to the PWWP and RPR domains.

**Methods S2: Analysis of *HULK* gene expression patterns**

*RNA-seq*

We used RNA-seq read data to assess the relative expression level of each *HULK* gene in roots, aerial seedlings and stage 12 floral bud tissues in each of 19 accessions that are parents of the Multiparent Advanced Generation InterCross genetic mapping resource ([Kover *et al.*, 2009](#_ENREF_8)). Briefly, RNA-seq reads for two of these accessions (Col-0 and Can-0) were previously published (Gene Expression Omnibus series number GSE30795), and analogous data for the remaining 17 accessions is released as part of this study (for methods, see ([Gan *et al.*, 2011](#_ENREF_6))). For each of the 57 accession and tissue combinations, RNA-seq reads were aligned to the Arabidopsis genome and TAIR10 annotation with TopHat 2.0.9 and Bowtie2 2.1.0 (parameters used for mapping were: -a 5 -i 5 -I 32000 --b2-very-sensitive --segment-mismatches 2), and Cufflinks 2.1.1 was used to calculate normalized gene expression levels using default parameters ([Trapnell *et al.*, 2012](#_ENREF_9)).

*In situ hybridization*

Additionally, *in situ* hybridization with gene-specific probes was used to assess spatial expression patterns. *HUA2* and *HULK1* specific cDNA probes were amplified using primers listed in Supplemental Table S6. The PCR products were cloned into the pGEM-T easy vector. To obtain the *HULK2* probe, a SpeI and PstI cDNA fragment was cloned into the pGEM-T easy vector. A *HULK3* specific PstI cDNA fragment was cloned in pGEM-T easy vector. Oligo-nucleotide primer sequences for PCR amplification of full length *HULK1, HULK2* and *HULK3* are listed in Supplemental Table S6. Finally, the RNA *in situ* hybridization was performed with *HULK* gene probes containing nucleotides 461-1357 relative to start codon for *HUA2*, 508-1122 for *HULK1*, 1675-2301 for *HULK2* and 1400-2046 for *HULK3*. Vegetative apices were collected from 28-days-old short day grown plants and inflorescence apices were collected from long day grown flowering plants. Non-radioactive RNA *in situ* hybridization was performed as previously described ([Weigel and Glazebrook, 2002](#_ENREF_10)).

*GUS staining*

For *HULK2::GUS,* 876 bp fragment of *HULK2* promoter was amplified with Expand High Fidelity Enzyme (Roche). The PCR product was cloned into pGEM-T Easy vector for sequence verification. The pGEM-T easy vector was digested with *EcoRI* enzyme. The digested product was gel purified and cloned in to the pRITA I vector. The pRITA I vector containing the *HULK2* promoter, GUS sequence and NOS terminator were excised with *NotI* and ligated into the pMLBart binary vector. The orientation of the insert was determined by restriction digestion with *XhoI.*

For *HULK3::GUS,* a 1203 bp fragment of *HULK3* promoter was amplified with Expand High Fidelity Enzyme (Roche). The PCR product was cloned into pGEM-T Easy vector for sequence verification. The pGEM-T easy vector was digested with *EcoRI* enzyme. The digested product was gel purified and cloned in to the pRITA I vector. The orientation of the insert in pRITA I was confirmed with *XhoI* digestion. The pRITA I vector containing the *HULK2* promoter, GUS sequence and NOS terminator were excised with *NotI* and ligated into pMLBart binary vector. The orientation of the insert was determined by restriction digestion with *HindIII.*

GUS staining was performed as previously described ([Weigel and Glazebrook, 2002](#_ENREF_10)). Primers used in cloning the *HULK* promoters are indicated in Supplemental Table S6.

**Methods S3: Expression profiling of *HULK* mutants**

*RNA collection and preparation of mRNA-seq libraries for HULK mutants*

Col-0, *hua2-7*, *hua2-7 hulk1*, and *hua2-7 hulk1 hulk2* seedling tissue, with biological replication, was collected for the production of Illumina mRNA sequencing (RNA-seq) libraries. Seeds were sterilized with chlorine gas for 3 hours, and stratified for 5 days in 0.1% agarose at 4°C. Plants were germinated and grown on Sunshine Mix 4 Aggregate Plus soil (cat. no. LA4, Sun Gro Horticulture, Bellevue, WA, USA) supplemented with ~0.03 g of Miracle-Grow® all-purpose plant food in 10 cm square pots in a Percival AR-66L environment growth chamber (see Gan et al., 2011 for growth details). All genotypes were grown at 20°C under long day growth conditions (16:8 hours light:dark). Approximately 20 seedlings were grown per pot, and 9 pots were planted per genotype. Plants were rotated through the chamber daily to eliminate potential environmental effects on development resulting from position within the growth chamber. To secure developmental uniformity of samples, the seedling tissues were collected when the 4th true leaf was visible. The aerial portions of seedlings were harvested by detachment just below the cotyledons. Twenty seedlings were collected and combined for each genotype and biological replicate. To minimize environmental effects, seedlings for a given replicate were collected in approximately equal numbers from each pot within the growth chamber for a given genotype. Biological replicates for each genotype were collected simultaneously. To eliminate circadian-dependent effects on gene expression (i.e., see ([Doherty and Kay, 2010](#_ENREF_3))), tissue collections were carried out at 8 ± 0.5 hours into the light cycle as described by ([Gan *et al.*, 2011](#_ENREF_6)). Tissue was collected directly into liquid nitrogen and subsequently stored at -80°C. Total RNA isolation and DNase treatment was performed as described previously ([Gan *et al.*, 2011](#_ENREF_6)). Messenger RNA (mRNA) was purified from 35 µg of total RNA using two rounds of selection with DynalBeads® Oligo (dT)_25_ beads (cat. no. 610.05, Invitrogen). Isolated mRNA was suspended in RNase-free water. To facilitate analysis of multiple RNA samples, with replication, mRNA was used to construct barcoded RNA-seq libraries with methods adapted from the Illumina mRNA sample preparation protocol (cat. no 1004894 Rev.A; ([Gan *et al.*, 2011](#_ENREF_6))). The concentration and size distribution of each library was evaluated on a Bioanalyzer 2100 with DNA 1000 Kits (Agilent, cat. no. 5067-1504); in all cases, the observed library sizes agreed closely with that expected from the size selection. Template concentrations were adjusted to 10 nM with a solution of 10 mM Tris-HCl, pH 8.5, containing 0.1% (v/v) Tween 20.

*Generation and quantification of RNA-seq reads for HULK mutants*

Illumina single-read cluster generation and sequencing was performed according to manufacturer’s instructions. Briefly, flow-cell preparation on a Cluster Station was performed with v5 Illumina Single Read Cluster Generation Kits (cat. no. GD-203-5001) using the “SR_Amplification_Linearization_Blocking_PrimerHyb_v7” Cluster Station workflow. For cluster generation, equal volumes of four barcoded 10 nM libraries were combined and diluted to 8 pM for flow-cell hybridization. All sequencing was performed on a Genome Analyzer IIx (GAIIx) instrument using v5 Sequencing Kits (cat. no. 15003925) and with an Illumina supported modification of the “GA2_76Cycle_SR_v7.xml” sequencing program to perform 82 cycles of imaging. We used version SCS2.6 of the GAIIx control software with Real Time Analysis (RTA) enabled and set to use “per lane” parameters for base-calling. RNA-seq reads were processed, quality filtered, trimmed and assigned to specific libraries using barcode sequences as described by ([Gan *et al.*, 2011](#_ENREF_6)) (resultant reads were 78 bp in length following barcode removal).

*Detection and analysis of differentially expressed genes from aligned RNA-seq reads*

RNA-seq read alignments to gene models and expression quantification were performed using the Bowtie/Tophat software package ([Trapnell *et al.*, 2012](#_ENREF_9)) and Python scripts. The following parameters were used for performing read alignment: -a 5, -i 5, -I 32000, --solexa1.3-quals, -g 1, --segment-mismatches 3, -p 5 with the TAIR10 annotation. To assess differential gene expression, we used the DESeq R package ([Anders and Huber, 2010](#_ENREF_1)). Raw read count data from alignments was used as input for DESeq and pair-wise comparisons were examined between Col-0 and each mutant genotype assayed (three comparisons in total: Col-0 vs. *hua2-7*, Col-0 vs. *hua2-7 hulk1,* and Col-0 vs. *hua2-7 hulk1 hulk2*). Genes with a FDR adjusted *P*-value < 0.05 by the Benjamini-Hochberg procedure, as implemented in DESeq, and greater than a 2-fold change were defined as differentially expressed for downstream analyses (Table S3). Gene Ontology enrichment analysis was carried out using the online GO analysis toolkit agriGO (<http://bioinfo.cau.edu.cn/agriGO/index.php>, ([Du *et al.*, 2010](#_ENREF_4))). Plant GOslim terms enriched in our gene set were identified using a Fisher exact test with significance 0.05 (Supplemental Table S4).

REFERENCES FOR SUPPORTING EXPERIMENTAL PROCEDURES

**Anders, S. and Huber, W.** (2010) Differential expression analysis for sequence count data. *Genome Biol*, **11**.

**Castresana, J.** (2000) Selection of conserved blocks from multiple alignments for their use in phylogenetic analysis. *Molecular Biology and Evolution*, **17**, 540-552.

**Doherty, C.J. and Kay, S.A.** (2010) Circadian control of global gene expression patterns. *Annu Rev Genet*, **44**, 419-444.

**Du, Z., Zhou, X., Ling, Y., Zhang, Z. and Su, Z.** (2010) agriGO: a GO analysis toolkit for the agricultural community. *Nucleic Acids Res*, **38**, 64-70.

**Edgar, R.C.** (2004) MUSCLE: multiple sequence alignment with high accuracy and high throughput. *Nucleic Acids Res*, **32**, 1792-1797.

**Gan, X., Stegle, O., Behr, J., Steffen, J.G., Drewe, P., Hildebrand, K.L., Lyngsoe, R., Schultheiss, S.J., Osborne, E.J., Sreedharan, V.T., Kahles, A., Bohnert, R., Jean, G., Derwent, P., Kersey, P., Belfield, E.J., Harberd, N.P., Kemen, E., Toomajian, C., Kover, P.X., Clark, R.M., Ratsch, G. and Mott, R.** (2011) Multiple reference genomes and transcriptomes for Arabidopsis thaliana. *Nature*, **477**, 419-423.

**Goodstein, D.M., Shu, S., Howson, R., Neupane, R., Hayes, R.D., Fazo, J., Mitros, T., Dirks, W., Hellsten, U., Putnam, N. and Rokhsar, D.S.** (2012) Phytozome: a comparative platform for green plant genomics. *Nucleic Acids Res*, **40**, 1178-1186.

**Kover, P.X., Valdar, W., Trakalo, J., Scarcelli, N., Ehrenreich, I.M., Purugganan, M.D., Durrant, C. and Mott, R.** (2009) A Multiparent Advanced Generation Inter-Cross to fine-map quantitative traits in Arabidopsis thaliana. *PLoS Genet*, **5**.

**Trapnell, C., Roberts, A., Goff, L., Pertea, G., Kim, D., Kelley, D.R., Pimentel, H., Salzberg, S.L., Rinn, J.L. and Pachter, L.** (2012) Differential gene and transcript expression analysis of RNA-seq experiments with TopHat and Cufflinks. *Nat Protoc*, **7**, 562-578.

**Weigel, D. and Glazebrook, J.** (2002) *Arabidopsis: a laboratory manual*: Cold Spring Harbor Laboratory, New York.
