# Supplementary material for: A plant-specific HUA2-LIKE (HULK) gene family in Arabidopsis thaliana is essential for development
Source: Plant J. 2014 Aug 28;80(2):242–54. doi: 10.1111/tpj.12629 (PMC4283595; doi:10.1111/tpj.12629)
Supplement: Supplementary file 22 [file tpj0080-0242-sd22.docx]

SUPPORTING INFORMATION LEGENDS

**Figure S1.** Protein alignment, conservation, consensus sequence and % similarity and identity matrix of the PWWP (**a**) and RPR (**b**) domains found in the Arabidopsis HULK proteins.

**Figure S2.** Rooted phylogram of amino acid sequences in domains of 69 HULK-like proteins of 28 Embryophyte species with *Physcomitrella patens* as an outgroup based on maximum likelihood. Sequences highlighted in purple: multiple HUA2-like proteins from species having representatives of only the HUA2/HULK1 or the HULK2/HULK3 clades; sequences highlighted in yellow: species represented by a single HUA2-like protein. The scale bar is a number of amino acid substitutions per site. Support values are puzzle support values.

**Figure S3.** Gene expression levels of *HUA2*, *HULK1*, *HULK2* and *HULK3* in various tissues and organs. (**a**) Data obtained from the developmental set of AtGenExpress for accession Col-0 (http://jsp.weigelworld.org/expviz/expviz.jsp, Schmid *et al*., 2005). (**b**) Mean levels of expression for each *HULK* gene as averaged from RNA-Seq data over each MAGIC founder accession for each of three tissues (*n* = 19 for each tissue; whole root of 10-day-old seedlings, aerial seedlings at emergence of the fourth true leaf, and stage 12 floral buds as indicated from left to right). FPKM is Fragments Per Kilobase of transcript per Million mapped reads. Error bars denote ± 2 standard errors of the mean.

**Figure S4.** *HULK2* and *HULK3* gene expression in embryo and pollen development as detected by *in situ* hybridization and GUS staining (**a**). Negative control for *HULK* gene expression in vegetative shoot and inflorescence apices as detected by *in situ* hybridization. Longitudinal sections of vegetative shoot (**b**) and inflorescence apices (**c**) hybridized with sense (s) probes to the four *HULK* genes as indicated. All sections are from wildtype (Col-0) plants. Scale bars: 35 μm in a, 100 μm in b and c.

**Figure S5.** *HUA2* and *HULK1-3* mutant alleles used in this study. (**a**) Position of T-DNA insertions in the *hua2-7*, *hulk1-1*, *hulk2-1* and *hulk3-1* alleles employed in this study. At each locus, arrows indicate the location and direction of primers used to assay expression by RT-qPCR. Exons are indicated by rectangles, introns by lines. (**b**) Mean ± standard error of the mean fold changes of *HULK1*, *HULK2* and *HULK3* relative to Col-0 in T-DNA insertion lines as assessed by RT-qPCR (*n* = 3). n.d., not detected.

**Figure S6.** Alignments of Illumina RNA-Seq reads to the *HUA2*, *HULK1* and *HULK2* loci in the *hua2-7*, *hulk1* and *hulk2* genetic backgrounds as indicated. Gene models are as shown at top (thick bars indicate coding sequences), and red arrows indicate positions of T-DNA insertions. Light blue lines indicate read alignments that span exons junctions. All aligned reads for a given genotype are shown. Normalized expression values (reads per million mapped, or RPM) for each *HULK* gene are given in Table S2.

**Figure S7.** Characterization of T2 *HULK1*-amiRNA and *HUA2/HULK1*-amiRNA plants. (**a**) Expression levels of *HULK1* and *HUA2* in 7-day old seedlings and leaves as determined by RT-qPCR. Values represent means of two replicates ± standard error of the mean. (**b**) Phenotypes of Col-0, *HULK1*-amiRNA and *hulk1* plants. (**c**) Plant and silique phenotypes in Col-0, *HUA2/HULK1*-amiRNA and *hua2-7 hulk1* lines. VC – plants transformed with empty vector. Scale bars: 1 cm in b and c (main figure), 1 mm in c (silique inset).

**Figure S8.** RT-qPCR analysis of the effect of amiRNA targeting *HULK2/HULK3* on levels of *FLC, HULK2* and *HULK3* in T1 Col-0 seedlings transformed with either *HULK2/HULK3-*amiRNA construct (labelled amiRNA, *n* = 20) or empty vector (labelled VC, *n* = 12). Mean ± standard error of the mean fold changes of *FLC*, *HULK2* and *HULK3*. Asterisks indicate significant differences in expression levels between treatments (Tukey HSD test, ** - *P* < 0.01, *** - *P* < 0.001).

**Figure S9.** Log_2_ transformed RNA-Seq gene expression levels (reads per million mapped) of biologically replicated seedling samples with genotypes as indicated. R^2^ values were calculated with all genes for which five or more reads mapped in at least one biological replicate.

**Figure S10.** Venn diagram (above) comparing and contrasting the top 20 GO functional categories (below) in the *hua2-7*, *hua2-7 hulk1* and *hua2-7 hulk1 hulk2* mutants. Genes used for the analysis were detected as differentially expressed by RNA-Seq profiling.

**Table S1.** Number of RNA-seq reads aligned per sample by genotype and biological replicate.

**Table S2**. Gene expression per TAIR10 gene model inferred from RNA-seq data for Col-0 and *HULK* mutant plants.

**Table S3.** Differentially expressed genes discovered by Illumina RNA-Seq *hua2-7*, *hua2-7 hullk1* and *hua2-7 hulk1 hulk2* relative to Col-0.

**Table S4.** Significantly represented (*P* < 0.05) Gene Ontology (GO) slim terms in sets of differentially expressed genes in *hua2-7*, *hua2-7 hullk1* and *hua2-7 hulk1 hulk2* relative to Col-0*.*

**Table S5.** Loss of function phenotypes of plants bearing mutations in DEGs identified by RNA-seq in single, double and triple mutants.

**Table S6**. List of putative HULK-like proteins used in this study.

**Table S7.** List of primers used in this study.

**Methods S1.** Preparation of dataset for phylogenetic analysis of HUA2-like sequences in Embryophytes.

**Methods S2.** Analysis of *HULK* gene expression patterns.

**Methods S3.** Expression profiling of *HULK* mutants.
